# Cryo-EM structure of the nucleosome core particle containing *Giardia lamblia* histones

**DOI:** 10.1093/nar/gkab644

**Published:** 2021-08-05

**Authors:** Shoko Sato, Yoshimasa Takizawa, Fumika Hoshikawa, Mariko Dacher, Hiroki Tanaka, Hiroaki Tachiwana, Tomoya Kujirai, Yukari Iikura, Cheng-Han Ho, Naruhiko Adachi, Indu Patwal, Andrew Flaus, Hitoshi Kurumizaka

**Affiliations:** Laboratory of Chromatin Structure and Function, Institute for Quantitative Biosciences, The University of Tokyo, 1-1-1 Yayoi, Bunkyo-ku, Tokyo 113-0032, Japan; Laboratory of Chromatin Structure and Function, Institute for Quantitative Biosciences, The University of Tokyo, 1-1-1 Yayoi, Bunkyo-ku, Tokyo 113-0032, Japan; Laboratory of Chromatin Structure and Function, Institute for Quantitative Biosciences, The University of Tokyo, 1-1-1 Yayoi, Bunkyo-ku, Tokyo 113-0032, Japan; Laboratory of Chromatin Structure and Function, Institute for Quantitative Biosciences, The University of Tokyo, 1-1-1 Yayoi, Bunkyo-ku, Tokyo 113-0032, Japan; Laboratory of Chromatin Structure and Function, Institute for Quantitative Biosciences, The University of Tokyo, 1-1-1 Yayoi, Bunkyo-ku, Tokyo 113-0032, Japan; Division of Cancer Biology, The Cancer Institute of Japanese Foundation for Cancer Research, 3-8-31 Ariake, Koto-ku, Tokyo, 135-8550, Japan; Laboratory of Chromatin Structure and Function, Institute for Quantitative Biosciences, The University of Tokyo, 1-1-1 Yayoi, Bunkyo-ku, Tokyo 113-0032, Japan; Laboratory of Chromatin Structure and Function, Institute for Quantitative Biosciences, The University of Tokyo, 1-1-1 Yayoi, Bunkyo-ku, Tokyo 113-0032, Japan; Laboratory of Chromatin Structure and Function, Institute for Quantitative Biosciences, The University of Tokyo, 1-1-1 Yayoi, Bunkyo-ku, Tokyo 113-0032, Japan; Department of Biological Sciences, Graduate School of Science, The University of Tokyo, Tokyo, Japan, 1-1-1 Yayoi, Bunkyo-ku, Tokyo 113-0032, Japan; Structural Biology Research Center, Institute of Materials Structure Science, High Energy Accelerator Research Organization (KEK), 1-1 Oho, Tsukuba, Ibaraki 305-0801, Japan; Center for Chromosome Biology, Biochemistry, School of Natural Sciences, National University of Ireland Galway, H91 TK33, Ireland; Center for Chromosome Biology, Biochemistry, School of Natural Sciences, National University of Ireland Galway, H91 TK33, Ireland; Laboratory of Chromatin Structure and Function, Institute for Quantitative Biosciences, The University of Tokyo, 1-1-1 Yayoi, Bunkyo-ku, Tokyo 113-0032, Japan; Department of Biological Sciences, Graduate School of Science, The University of Tokyo, Tokyo, Japan, 1-1-1 Yayoi, Bunkyo-ku, Tokyo 113-0032, Japan

## Abstract

*Giardia lamblia* is a pathogenic unicellular eukaryotic parasite that causes giardiasis. Its genome encodes the canonical histones H2A, H2B, H3, and H4, which share low amino acid sequence identity with their human orthologues. We determined the structure of the *G. lamblia* nucleosome core particle (NCP) at 3.6 Å resolution by cryo-electron microscopy. *G. lamblia* histones form a characteristic NCP, in which the visible 125 base-pair region of the DNA is wrapped in a left-handed supercoil. The acidic patch on the *G. lamblia* octamer is deeper, due to an insertion extending the H2B α1 helix and L1 loop, and thus cannot bind the LANA acidic patch binding peptide. The DNA and histone regions near the DNA entry-exit sites could not be assigned, suggesting that these regions are asymmetrically flexible in the *G. lamblia* NCP. Characterization by thermal unfolding in solution revealed that both the H2A–H2B and DNA association with the *G. lamblia* H3–H4 were weaker than those for human H3–H4. These results demonstrate the uniformity of the histone octamer as the organizing platform for eukaryotic chromatin, but also illustrate the unrecognized capability for large scale sequence variations that enable the adaptability of histone octamer surfaces and confer internal stability.

## Graphical Abstract

**Graphical Abstract:**
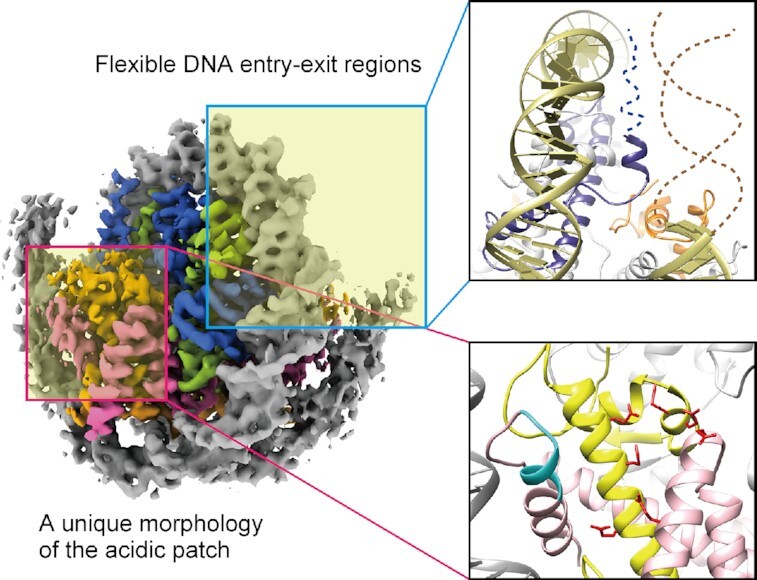
*G. lamblia* has an equivalent nucleosome structure, despite low histone sequence identity to metazoan nucleosomes. The DNA entry-exit and acidic patch regions are unique characteristics of *G. lamblia* nucleosomes.

## INTRODUCTION

In the nucleus of eukaryotic cells, genomic DNA is packaged into chromatin as a crucial structure for the maintenance of genome organization. Chromatin stability and conformational dynamics play an essential role in regulating biological events, such as gene transcription, DNA replication and DNA repair (1). The nucleosome consists of the nucleosome core particle (NCP) and linker DNAs, as the fundamental structural unit of chromatin. The biophysical properties of the nucleosome are major factors influencing the higher-order chromatin conformation (2–4).

The NCP is composed of 145–147 base-pairs of DNA wrapped around a histone octamer, containing two molecules each of the four histone proteins, H2A, H2B, H3 and H4 (5–9). These histone proteins share two characteristic motifs. One is the histone fold domain, which consists of three α-helices separated by two loops. The H2A–H2B dimer and the H3–H4 tetramer are assembled via packing of the histone fold domains, and two H2A–H2B dimers associate with one H3–H4 tetramer to form the histone octamer at the core of the NCP (10). The other motif consists of the flexible histone N- and C-terminal tails that extend outward from the NCP, with many residues targeted by histone modifying enzymes and other nuclear proteins (11,12). The overall NCP configuration is considered to be highly conserved across eukaryotic species. In histones, specific amino acid residues affect their biophysical properties, such as nucleosome stability, positioning, and flexibility of the nucleosomal DNA, through alternative DNA-histone and histone-histone interactions, and hence these in turn influence the chromatin dynamics (13). This has led to a common assumption that histones are amongst the most highly conserved proteins in eukaryotes.

*Giardia lamblia* is a unicellular eukaryotic microorganism classified as a diplomonad that parasitically infects the small intestines of humans and other vertebrates to cause giardiasis, one of the most common waterborne diarrheal diseases throughout the world (14,15). *Giardia* species exist in two forms during their life cycle: The vegetative or trophozoite form causes the primary symptoms of diarrhea by proliferating on the surface of the host intestinal cells. In contrast, the highly infective cyst form is environmentally resistant and able to survive outside of the host for long periods of time (16). Because of its simple cellular organization, *G. lamblia* is a useful model organism for key biological processes characteristic of the eukaryotic cell (17,18). *G. lamblia* is also regarded as an interesting organism in eukaryotic evolution because of its biological simplicity, and the evolutionary status of this organism, resulting from early divergence or reductive evolution, is currently debated (17,18).

*G. lamblia* contains a 12-megabase-pair gene-rich genome (19). Genes encoding the canonical histones H2A, H2B, H3 and H4, as well as the histone variants H3B and cenH3, have been identified and are encoded without introns, as in most other eukaryotes (20–24). Histone modifications such as acetylation, methylation, and ubiquitination have been detected, suggesting that epigenetic genome regulation may occur (25). Several critical processes for the parasite, such as encystation, are likely to be regulated in an epigenetic manner (26–29).

The histones of parasitic protozoa are generally evolutionally diversified, with lower identity to metazoan histones than other eukaryotes (22,30). Even among protozoans, the *Giardia* parasite stands out as having remarkably low histone identity with human histones (41–70%). The structural and biophysical implications of such large histone sequence variations in the *Giardia* nucleosome have not been investigated. In the present study, we determined the cryo-electron microscopy (cryo-EM) structure of the *G. lamblia* NCP at 3.6 Å resolution, and characterized the stabilities of the nucleosome and histone octamer in solution. Our studies revealed a number of structural features that can vary while still retaining the characteristic fold and DNA wrapping of the nucleosome.

## MATERIALS AND METHODS

### Histone purification

The DNA fragments encoding the *G. lamblia* histones H2A, H2B, H3, H4, and H2A*^G.lamblia^*^CTD^ were cloned into the pET-15b vector, which encodes the hexahistidine (His_6_)-tag and thrombin protease recognition sequences. The *G. lamblia* histones H2A, H2B, H3 and H2A*^G.lamblia^*^CTD^ were produced in *Escherichia coli* BL21 (DE3) cells, and the *G. lamblia* histone H4 was produced in *Escherichia coli* Rosetta-gami B (DE3) cells, by induction with 0.5 mM isopropyl-β-D-thiogalactopyranoside. The cells were cultured overnight at 37°C. The *G. lamblia* histones H2A, H2B, H3, and H2A*^G.lamblia^*^CTD^ were purified by the same method used for the purification of human histones, as described previously (31). The *G. lamblia* histone H4 was purified by a slightly modified method. Briefly, the *G. lamblia* histone H4 was purified by nickel-nitriloacetic acid (Ni-NTA) agarose column chromatography (QIAGEN) with a linear gradient of 5 mM to 1 M imidazole in 50 mM Tris–HCl buffer (pH 8.0), containing 0.5 M NaCl, 6 M urea, and 5% glycerol. The eluted samples were dialyzed against  50 mM Tris–HCl buffer (pH 7.5), containing  2 mM 2-mercaptoethanol. The N-terminal His_6_ tag was removed from the histone H4 portion by thrombin protease treatment. Afterwards, the histone H4 without the His_6_ tag was subjected to MonoS column chromatography (Cytiva). The column was washed with  20 mM sodium acetate buffer (pH 5.2), containing 0.2 M NaCl, 5 mM 2-mercaptoethanol, 1 mM EDTA and  6 M urea, and the histone H4 was eluted by a linear gradient of 0.2–1.2 M NaCl in 20 mM sodium acetate buffer (pH 5.2), containing 5 mM 2-mercaptoethanol, 1 mM EDTA and 6 M urea. All purified histones were dialyzed against water containing 2 mM 2-mercaptoethanol, and then freeze-dried.

### Histone octamer, tetramer, and dimer reconstitution and purification

For the histone octamer reconstitution, the *G. lamblia* or human histones H2A, H2B, H3 and H4, or H2A*^G.lamblia^*^CTD^, human histones H2B, H3 and H4 were mixed at a 1:1:1:1 molar ratio in 50 mM Tris–HCl buffer (pH 7.5), containing 7 M guanidine hydrochloride and 20 mM 2-mercaptoethanol. For the histone H3–H4 tetramer and H2A–H2B dimer reconstitution, the *G. lamblia* or human histones H3 and H4, or H2A and H2B, were mixed at a 1:1 molar ratio, respectively, in 50 mM Tris–HCl buffer (pH 7.5), containing 7 M guanidine hydrochloride and 20 mM 2-mercaptoethanol. The histone octamer, tetramer, and dimer were assembled by dialysis against 10 mM Tris–HCl buffer (pH 7.5), containing 2 M NaCl, 1 mM EDTA and 5 mM 2-mercaptoethanol, and then purified by size exclusion chromatography on a Hiload 16/60 Superdex 200 prep grade column (Cytiva).

### NCP and H3–H4–DNA complex reconstitution

DNA fragments containing a 145 base-pair modified palindromic 601 (601L) sequence or a 146 base-pair palindromic human α-satellite sequence were prepared (5,9). For the NCP and the H3–H4–DNA complex reconstitution with the *G. lamblia* or human histones, the DNA fragments were mixed with a histone octamer or the H3–H4 tetramer in 10 mM Tris–HCl buffer (pH 7.5), containing 2 M KCl, 1 mM EDTA and 1 mM dithiothreitol. For the reconstitution of the NCPs containing the *G. lamblia* H3–H4 and the human H2A–H2B or the *G. lamblia* H2A–H2B and the human H3–H4, the DNA fragment containing a palindromic human α-satellite sequence was mixed with the *G. lamblia* H3–H4 tetramer and the human H2A–H2B dimer, or the *G. lamblia* H2A–H2B dimer and the human H3–H4 tetramer, in 10 mM Tris–HCl buffer (pH 7.5), containing 2 M KCl, 1 mM EDTA and 1 mM dithiothreitol. The NCPs and the H3–H4–DNA complexes were reconstituted by the salt-dialysis method, with the KCl concentration gradually decreased to 250 mM (31,32). The NCPs and the H3–H4–DNA complexes containing the palindromic human α-satellite DNA were heated at 55°C for 2 h. The reconstituted NCPs and the H3–H4–DNA complexes were purified by non-denaturing polyacrylamide gel electrophoresis using a Prep Cell apparatus (Bio-Rad), as described previously (31,32). The concentrations of the NCP and the H3–H4–DNA complex were estimated from the absorbance at 260 nm.

### Sample preparation for the cryo-EM analysis

The *G. lamblia* NCP containing the 601L sequence was stabilized by the GraFix method, using paraformaldehyde (33). A gradient was formed with solution 1 (10 mM HEPES–NaOH (pH 7.5), 20 mM NaCl, 1 mM dithiothreitol, and 5% sucrose) and solution 2 (10 mM HEPES–NaOH (pH 7.5), 20 mM NaCl, 1 mM dithiothreitol, 25% sucrose and 4% paraformaldehyde), using a Gradient Master 108 (BioComp instruments). The *G. lamblia* NCP was applied to the top of the gradient, which was then centrifuged at 27 000 rpm in a Beckman SW41Ti rotor. After centrifugation at 4°C for 16 h, fractions (0.65 ml) were successively collected from the top of the gradient. The absorbance of the fractions at 260 nm was monitored, and the peak fractions were collected. Using a PD-10 column (Cytiva), the buffer was exchanged to 20 mM HEPES–KOH (pH 7.5) buffer, containing 0.1 mM Tris–HCl (pH 8.0), 50 mM potassium acetate, and 0.2 μM zinc acetate. The resulting sample was concentrated with an Amicon Ultra centrifugal filter. The sample concentration was adjusted to 0.8 mg/ml for DNA. For the cryo-EM specimen preparation, a 2.5 μl sample was applied onto a Quantifoil R1.2/1.3 200-mesh Cu grid, which was treated with a PIB-10 glow discharge device (Vacuum Device, Japan). The grids were blotted for 8 s under 100% humidity at 16°C in a Vitrobot Mark IV (Thermo Fisher Scientific), and then immediately plunged into liquid ethane. The cryo-EM data collection conditions were screened with the Talos Arctica electron microscope (Thermo Fisher Scientific) installed at KEK.

### Cryo-EM data collection

The vitrified samples of the *G. lamblia* NCP were imaged at 300 kV on a Krios G3i microscope (Thermo Fisher Scientific), equipped with an energy-filtered K3 detector calibrated at a pixel size of 1.05 Å with a slit width of 25 eV, using the SerialEM auto acquisition software (34). The images of the *G. lamblia* NCP were recorded with a 5.6 second exposure time in the electron counting mode with a 140 milliseconds frame time, retaining a stack of 40 frames with an accumulated total dose of ∼64 electrons/Å^2^.

### Image processing

All frames of 8722 movies were aligned by MOTIONCOR2 (35) with dose weighting. The contrast transfer function (CTF) was estimated using CTFFIND4 (36). RELION 3.0 was used for the following image processing of the *G. lamblia* NCP (37). From 6429 micrographs, 2 133 462 particles were picked automatically, followed by two rounds of 2D classification, resulting in the selection of 1 611 159 particles. The 3D classification was performed using the initial model generated by RELION. Subsequently, 194 041 particles were subjected to the 3D refinement, followed by particle polishing and two rounds of CTF refinement. The final resolution of the refined *G. lamblia* NCP map was 3.57 Å, as estimated by the gold standard Fourier Shell Correlation (FSC) with the 0.143 criterion (38). The final map of the *G. lamblia* NCP was normalized with MAPMAN (39), and visualized with UCSF Chimera (40) and UCSF ChimeraX (41). The details of the processing statistics for the *G. lamblia* NCP are provided in Table 1.

**Table 1.** Cryo-EM data collection, processing, refinement and validation statistics

Sample	*G. lamblia* NCP (EMD-30591, PDB ID: 7D69)
**Data collection**	
Electron microscope	Krios G3i
Camera	K3
Pixel size (Å/pix)	1.05
Defocus range (μm)	–1.0 to –2.5
Exposure time (second)	5.6
Total dose (e/Å^2^)	64
Movie frames (no.)	40
Total micrographs (no.)	8722
**Reconstruction**	
Software	Relion 3.0
Particles for 2D classification	2 133 462
Particles for 3D classification	1 611 159
Particles in the final map (no.)	194 041
Symmetry	C1
Final resolution (Å)	3.57
FSC threshold	0.143
Map sharpening B factor (Å^2^)	–120
**Model building**	
Software	Coot
**Refinement**	
Software	Phenix
**Model composition**	
Protein	727
Nucleotide	250
**Validation**	
MolProbity score	2.15
Clash score	19.66
R.m.s. deviations	
Bond lengths (Å)	0.009
Bond angles (°)	0.875
**Ramachandran plot**	
Favored (%)	94.80
Allowed (%)	5.20
Outliers (%)	0.00

### Model building and refinement

The crystal structures of the nucleosomal human histones (PDB ID: 6R93) and 601L DNA (PDB ID: 3UT9) were placed in the cryo-EM map of the *G. lamblia* NCP, by rigid-body fitting with UCSF Chimera (40). The amino acid sequences were replaced with those of the *G. lamblia* histones, according to the sequence alignment by Clustal Omega (42), and the complete model of the *G. lamblia* NCP was manually built with COOT (43) followed by real-space refinement in Phenix (44). The *G. lamblia* NCP model and the human NCP model were visualized with UCSF Chimera and PyMOL (The PyMOL Molecular Graphics System, Version 2.0 Schrödinger, LLC).

### Micrococcal nuclease susceptibility assay

The reconstituted NCPs containing the palindromic human α-satellite or 601L sequence (1.2 μg for DNA) were incubated with 0.6 or 0.4 units of MNase (Takara, Japan), respectively, at 37°C, in a reaction solution containing 40 mM Tris–HCl (pH 8.0), 25 mM NaCl, 2.5 mM CaCl_2_ and 1.9 mM dithiothreitol. The reactions were stopped by adding an aliquot (10 μl) to 5 μl of a deproteinization solution, consisting of 20 mM Tris–HCl (pH 8.0), 20 mM EDTA, 0.1% SDS and 0.5 mg/ml proteinase K (Roche). The reaction products were analyzed by non-denaturing polyacrylamide gel electrophoresis, followed by ethidium bromide staining. Three experiments were independently performed, and the reproducibility was confirmed.

### Electrophoretic mobility shift assay

The DNA fragment encoding residues 1–23 of the viral latency-associated nuclear antigen (LANA) was ligated just downstream of the PreScission recognition sequence in the pGEX6P1 plasmid. The LANA peptide was produced as a fusion protein with GST (GST-LANA). The GST-LANA and GST proteins were produced in *E. coli* BL21(DE3) cells, and purified by Glutathione Sepharose 4B (Cytiva) column chromatography. The *G. lamblia* and human canonical NCPs containing the 601L DNA fragments were mixed with the purified GST-LANA or GST in a solution containing 14 mM Tris–HCl (pH 7.5), 4 mM HEPES–NaOH (pH 7.5), 0.45 mM EDTA, 150 mM NaCl, 0.8 mM dithiothreitol, and 4% glycerol. The LANA–NCP complexes were analyzed by 6% non-denaturing PAGE in 0.5× TBE (45 mM Tris base, 45 mM boric acid and 1 mM EDTA) buffer, followed by ethidium bromide staining.

### Analytical ultracentrifugation sedimentation velocity assay

The DNA fragment containing twelve tandem repeats of a 177 base-pair 601 sequence was prepared as described previously (45). The DNA fragments were mixed with a histone octamer in 10 mM Tris–HCl buffer (pH 7.5), containing 2 M NaCl and 0.1 mM EDTA, and the nucleosome arrays were reconstituted by the salt-dialysis method, in which the NaCl concentration was gradually decreased to 250 mM (46). The nucleosome arrays were dialyzed against 10 mM Tris–HCl buffer (pH 7.5). The nucleosome occupancy of the 601 sequence was evaluated by digestion with the *Sca*I restriction enzyme (Takara), which cleaves the linker DNA regions of the nucleosome arrays, as previously described (46). Briefly, nucleosome samples (100 ng of DNA) were digested by 10 units of *Sca*I in a 10 μl reaction solution (10 mM Tris–HCl (pH 7.5), 50 mM NaCl, 0.5 mM MgCl_2_ and 0.1 mg/ml BSA) at 22°C for 12 h, and the resulting mononucleosomes were analyzed by electrophoresis on a non-denaturing 5% polyacylamide gel in 1× TBE buffer (90 mM Tris base, 90 mM boric acid and 2 mM EDTA), followed by ethidium bromide staining. The gel image was acquired with an Amersham imager (Cytiva). The sedimentation velocity analysis of the nucleosome arrays (35 ng/μl of DNA) was performed using a Beckman Coulter ProteomeLab XL-I centrifuge with an 8-hole An-50Ti Analytical Rotor, at 22 000 rpm for 3 h in 10 mM Tris–HCl (pH 7.5). The collected data were analyzed with the SEDFIT software (47). The sedimentation coefficient was calculated with a partial specific volume of 0.65 cm^3^/g.

### Thermal stability assay

The stabilities of the NCPs containing the palindromic human α-satellite sequence were evaluated by a thermal stability assay, as described previously (48). The NCP (1.2 μM) or the H3–H4–DNA complex (1.2 μM) was incubated in a reaction solution, containing 20 mM Tris–HCl (pH 7.5), 100 mM NaCl, 1 mM dithiothreitol, and 1000-fold diluted SYPRO Orange Protein Gel Stain (Sigma-Aldrich). The temperature was increased from 25°C to 95°C, in steps of 1°C/min, and the SYPRO Orange fluorescence was detected using a StepOnePlus™ Real-Time PCR system (Applied Biosystems). The fluorescence intensities were normalized as follows: *F*(*T*)_normalized_ = [*F*(*T*) – *F*(26)]/[*F*(95) – *F*(26)]. *F*(*T*) indicates the fluorescence intensity at a particular temperature. The averages of three independent experiments were plotted with standard deviation values. The thermal stabilities of the histone octamers were evaluated as described previously (48). The histone octamer (2 μM) was incubated in a solution, containing 20 mM Tris–HCl (pH 7.5), 2 M NaCl, 1 mM dithiothreitol and 1000-fold diluted SYPRO Orange Protein Gel Stain. The thermal stability assay was performed by the same method used for the NCP. The fluorescence intensities were plotted against the temperatures.

## RESULTS AND DISCUSSION

### Cryo-EM structure of the *G. lamblia* NCP

**Figure 1.**
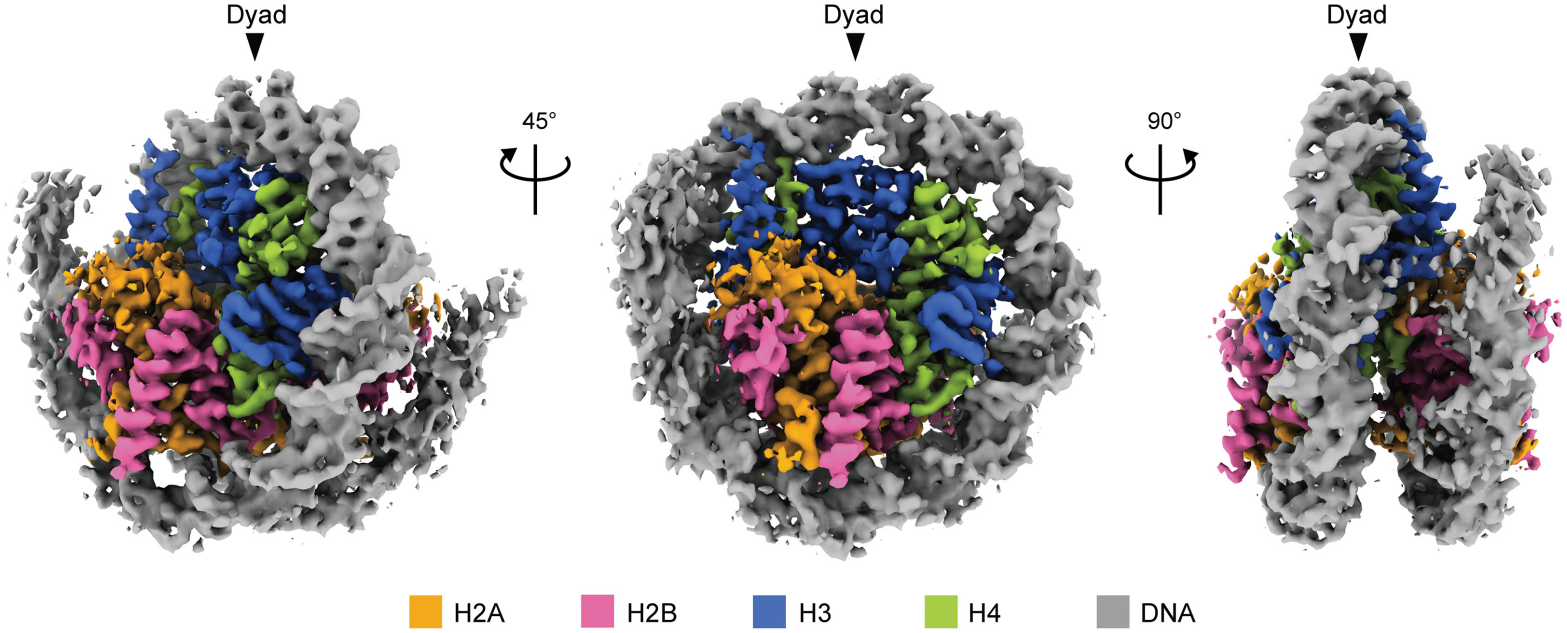
Cryo-EM structure of the *G. lamblia* NCP. The cryo-EM density map of the *G. lamblia* NCP. Histones H2A, H2B, H3 and H4 are colored orange, magenta, blue, and green, respectively and the DNA is colored gray. The cryo-EM map was visualized with the UCSF ChimeraX software. Arrowheads indicate the dyad axis of the *G. lamblia* NCP.

**Figure 2.**
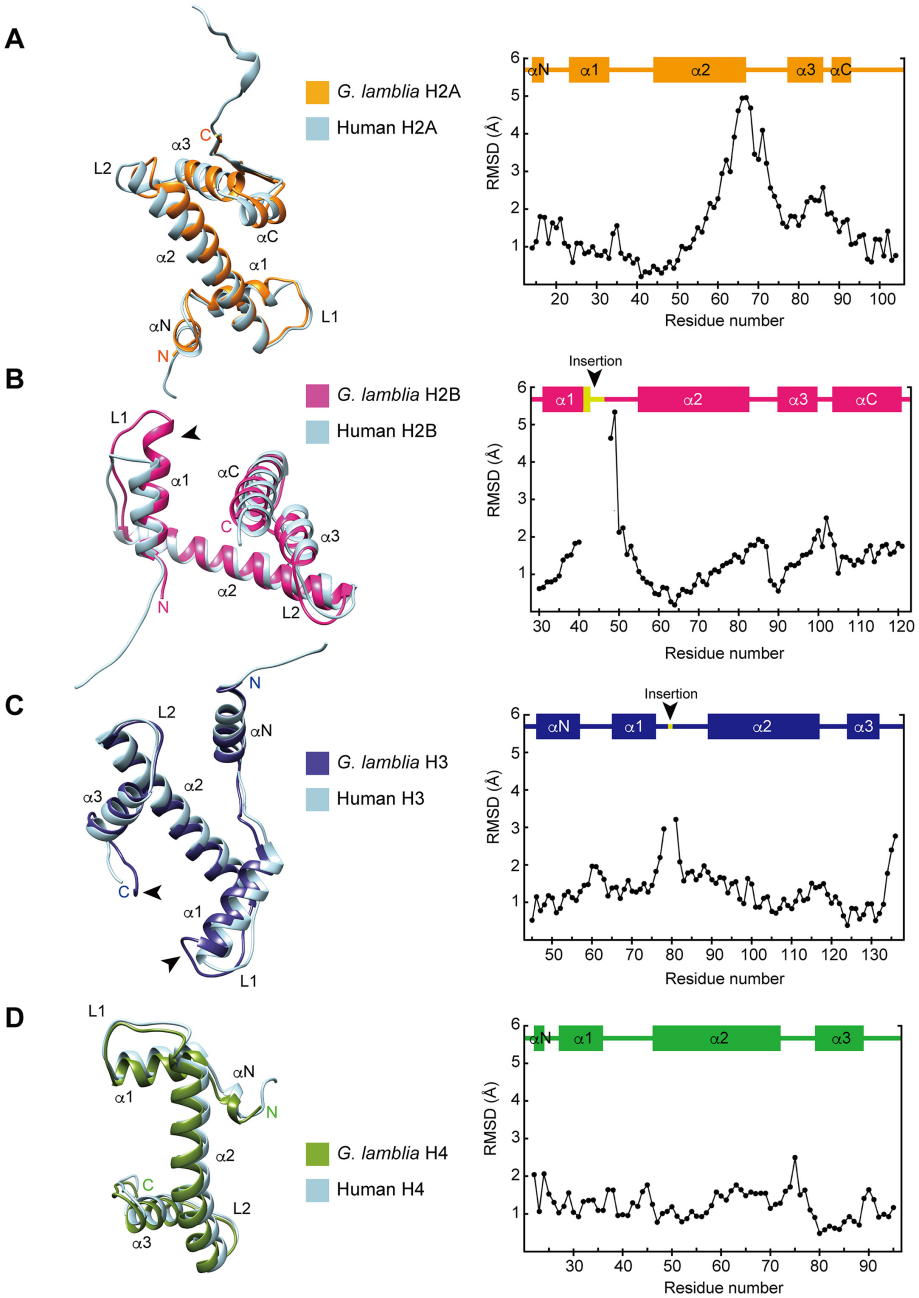
Histone structures in the *G. lamblia* NCP. Structural superimpositions of the nucleosomal histones H2A (**A**), H2B (**B**), H3 (**C**) and H4 (**D**) between the *G. lamblia* NCP and the human canonical NCP cryo-EM structure (PDB ID: 6R93). The *G. lamblia* and human NCP models were aligned in PyMOL and visualized with the UCSF Chimera software. *G. lamblia* histones H2A, H2B, H3 and H4 are colored orange, magenta, dark blue, and green, respectively, with human histones in light blue. Arrowheads on H2B and H3 represent the locations of the *G. lamblia*-specific insertions. RMSD values for aligned residue α-carbons are plotted in the right panels, with secondary structures above.

We reconstituted the NCP with recombinantly expressed *G. lamblia* histones (Supplementary Figure S1A, lane 1). The purified *G. lamblia* histones, H2A, H2B, H3, and H4, efficiently formed the NCP, in which the four histones were incorporated in a 1:1:1:1 stoichiometry (Supplementary Figure S1B, lane 1). The reconstituted *G. lamblia* NCP was fixed by sucrose gradient centrifugation with paraformaldehyde (GraFix; 33) (Supplementary Figure S1A and B, lane 2). We then visualized the cryo-EM structure of the *G. lamblia* NCP at 3.57 Å resolution by a single-particle workflow in the RELION software package (49) (Supplementary Figure S2 and Table 1). The overall structure of the *G. lamblia* NCP is similar to that of the canonical NCP (Figure 1 and Supplementary Figure S3). All *G. lamblia* histones adopted the characteristic histone fold domain, but with notable deviations from the backbone human histones in the NCP, including the *G. lamblia*-specific insertions of six and two residues in H2B and H3, respectively (Figure 2A–D with insertions shown by arrowheads, and Supplementary Figure S4). The root mean square deviations (RMSDs) of the *G. lamblia* histone fold domains in the NCP were 1.35–1.95 Å, as compared to canonical human histones in a cryo-EM structure (50) (Figure 2A–D and Table 2). For comparison, the RMSDs of the histone fold domains between the *Saccharomyces cerevisiae* (PDB ID: 1ID3) and human (PDB ID: 6R93) NCPs are 0.49–0.85 Å. A 125 base-pair DNA region (out of 145 bp) was wrapped around the histone octamer in a left-handed supercoil, with a mean RMSD of 2.01 Å from the canonical NCP with the same DNA sequence (9) (Supplementary Figure S3).

**Table 2.** RMSD values of the histones in the NCP

	Chain ID	RMSD of the histones in the NCP
H2A	Chain C*	1.95
	Chain G	1.80
H2B	Chain D*	1.73
	Chain H	1.91
H3	Chain A	1.58
	Chain E*	1.55
H4	Chain B	1.59
	Chain F*	1.35

The RMSD values were calculated from the distances between the α-carbons of the *G. lamblia* histones and the corresponding α-carbons of the human histones (PDB ID: 6R93) between the aligned *G. lamblia* and human NCPs, using the PyMOL software. Chains A–H correspond to the chains in the NCP structures deposited in the PDB (PDB IDs: 7D69 and 6R93). *The histone structures in the NCPs are shown in Figure 2.

### The flexible DNA ends of the *G. lamblia* NCP

**Figure 3.**
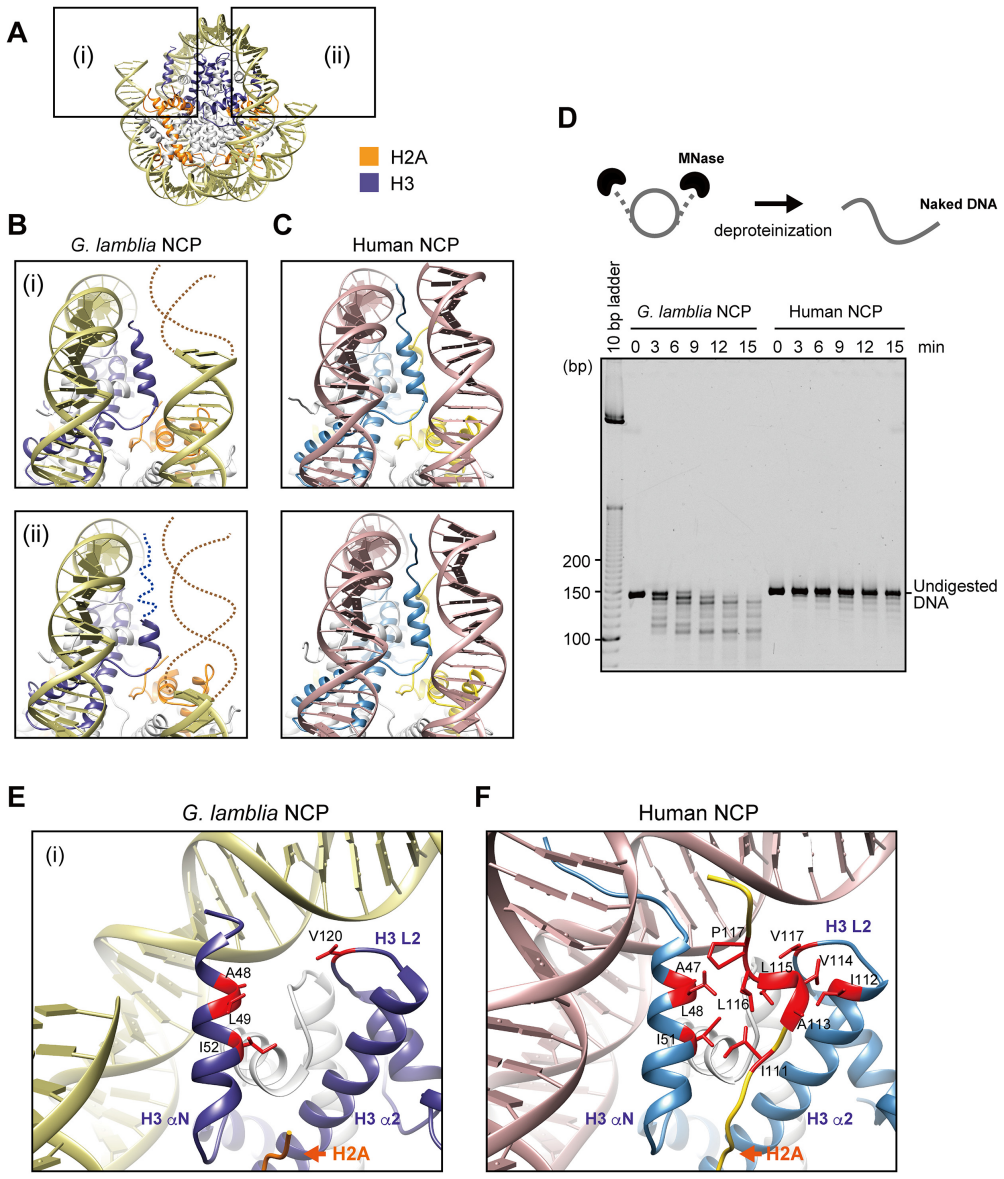
DNA flexibility around the entry-exit regions of the *G. lamblia* NCP. (**A**) The entry-exit DNA regions of the *G. lamblia* NCP are enclosed in boxes (i) and (ii). (**B**) Close-up views of (i) and (ii) from panel (A) with H2A and H3 in orange and blue, respectively. Dashed lines represent predicted DNA and H3 αN helix regions that are not visible in the *G. lamblia* NCP structure. (**C**) Human canonical NCP regions (PDB ID: 6R93) corresponding to the *G. lamblia* NCP regions shown in panel (B) with H2A and H3 are colored yellow and blue, respectively. (**D**) Schematic representation of the MNase sensitivity assay (upper panel). The NCPs containing palindromic human α-satellite DNA were treated with MNase. After MNase treatment of the *G. lamblia* and human canonical NCPs for the indicated times, DNA fragments were deproteinized and analyzed by non-denaturing polyacrylamide gel electrophoresis with ethidium bromide staining (lower panel). Additional independently performed experiments are shown in Supplementary Figure S5. (**E**) Close-up view around the visible H3 αN helix region of the *G. lamblia* NCP. (**F**) Close-up view of the human canonical NCP region corresponding to panel (E). Clustered hydrophobic H2A and H3 residues with sidechains are colored red.

The DNA ends of the *G. lamblia* NCP could not be resolved, whereas these regions are well defined in the structures of canonical NCPs (Figure 3A–C), suggesting that the entry-exit DNA regions of the *G. lamblia* NCP are more flexible and accessible. We performed a micrococcal nuclease (MNase) assay to test whether these DNA regions are differentially accessible in solution, since MNase preferentially accesses and digests the DNA regions detached from the histone surfaces in the NCP. The DNA ends of the *G. lamblia* NCP were substantially more susceptible to MNase than those of the control human NCP (Figure 3D and Supplementary Figure S5), in agreement with the *G. lamblia* NCP structure. We employed the GraFix method with paraformaldehyde in the cryo-EM sample preparation, and confirmed that this procedure did not enhance the DNA end detachment in the *G. lamblia* NCP (Supplementary Figure S1C).

The eight additional residues representing two turns of the H3 αN helix are also not resolved (Figure 3B). A similar shortened αN helix has been observed in the CENP-A NCP (51–54), where CENP-A is the centromeric histone H3 variant that identifies the centromere region in chromosomes (55–57). The positively charged human H3 residues Arg49 and Arg53 interact with the DNA around the entry/exit region of the NCP. These are replaced by lysine in both CENP-A and *G. lamblia* H3 (Supplementary Figure S4). A previous molecular dynamics simulation analysis and nuclease susceptibility assay suggested that the Arg49Lys and Arg53Lys substitutions may contribute directly to the DNA end flexibility in the CENP-A NCP (58), so the equivalent Lys50 and Lys54 (and/or Lys53) residues may play similar important roles in the DNA end flexibility in the *G. lamblia* NCP.

We also noticed that the lengths of the DNA ends of the *G. lamblia* NCP that could not be resolved were asymmetric (Figure 3B), even though the 601L DNA is palindromic and the assembled histones have full dyad symmetry. Asymmetry of the nucleosomal DNA end flexibility has been reported in the cryo-EM structures of CENP-A NCPs (59), in which non-palindromic DNA was employed. However, DNA end asymmetry was also found in the *G. lamblia* NCP with a symmetric palindromic DNA. Therefore, the asymmetric DNA end flexibility may be induced by a DNA sequence-independent mechanism. This was not previously tested with the CENP-A NCP. Notably, such non-equivalent histone-DNA interactions have been proposed for the allosteric characteristics, in which the DNA dissociation at the one end may stabilize the histone-DNA interactions at the opposite end in the nucleosome (60,61).

The N-terminal region of H3 directly binds to the DNA near the entry-exit regions and interacts with the C-terminal region of H2A in the human canonical NCP (Figure 3F). These H3–DNA and H3–H2A interactions are not observed in the *G. lamblia* NCP, probably due to the flexible nature of these regions (Figure 3E). The H2A C-terminal hydrophobic residues from 111–117 are important for the H3 binding in the human canonical NCP, and these are replaced by hydrophilic residues, including four charged sidechains, from 108–114 in *G. lamblia* H2A (Figure 3E and F, and Supplementary Figure S4). This appears to weaken the binding of the H2A C-terminal region to the H3 αN helix, and thus the H2A C-terminal region is not resolved on either face of the structure. Mutations of *Saccharomyces cerevisiae* H2A residues in this patch confer a Swi/Snf-independent (SIN) phenotype, and the mutagenesis of H2A Leu115 or Leu116 in metazoan histones affects nucleosome stability *in vitro* (62,63).

**Figure 4.**
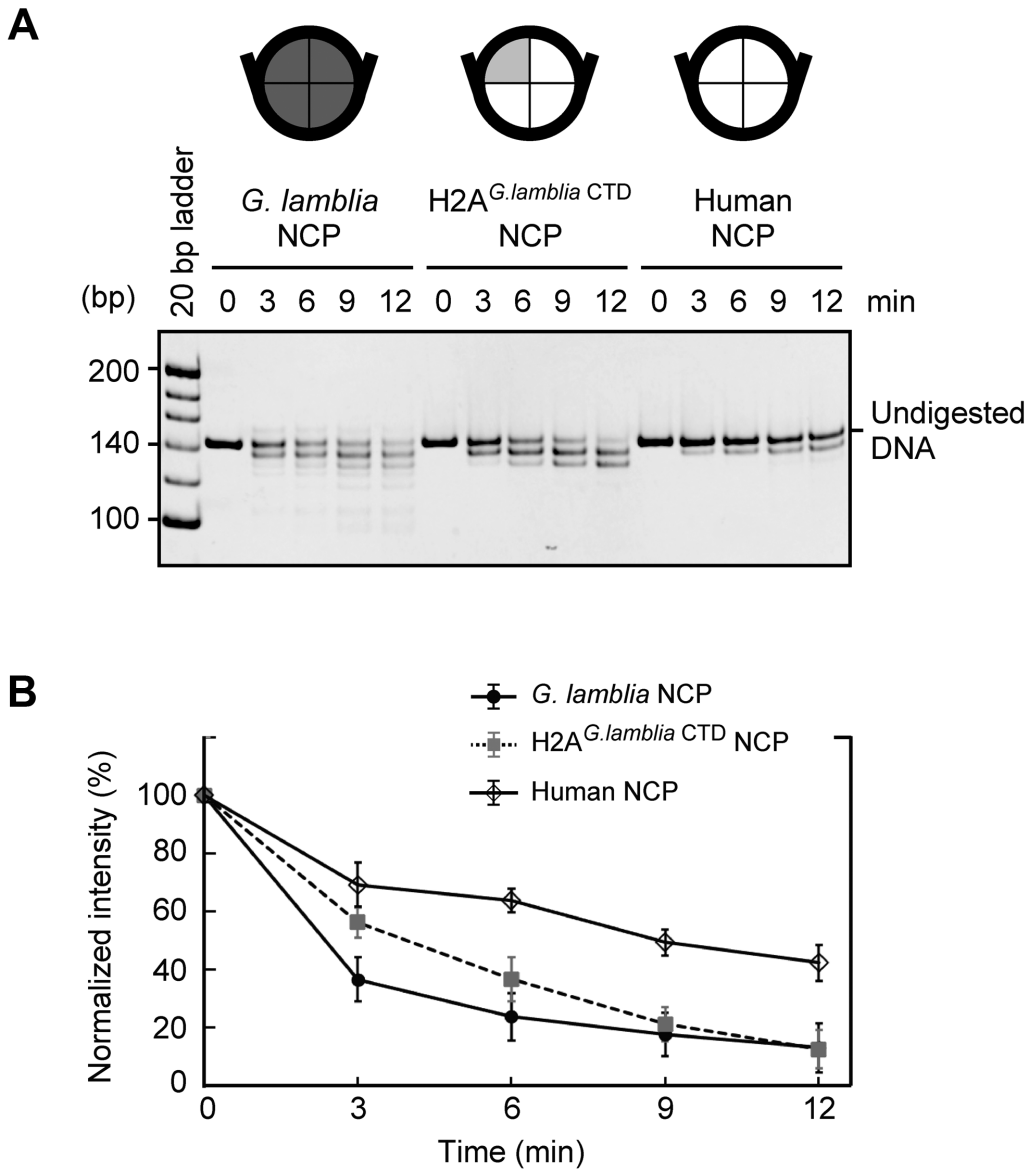
The C-terminal domain of the *G. lamblia* H2A contributes to the nucleosomal DNA end flexibility. (**A**) The *G. lamblia* NCP, the H2A*^G.lamblia^*^CTD^ NCP, and the human canonical NCP were reconstituted with the 601L DNA and treated with MNase for the indicated times. The DNA fragments were deproteinized and analyzed by non-denaturing polyacrylamide gel electrophoresis with ethidium bromide staining. The experiments were independently repeated three times, and the reproduced results are shown in Supplementary Figure S6. (**B**) Quantitation of the MNase assay. Undigested DNA was quantitated from stained gel images and plotted as mean and standard deviation (*n* = 3).

To test whether the C-terminal residues of the *G. lamblia* H2A also contribute significantly to the DNA end flexibility, we prepared a swapping mutant, H2A*^G.lamblia^*^CTD^, in which the human H2A C-terminal 20 residues are replaced with the corresponding *G. lamblia* H2A C-terminal 17 residues. The NCP containing H2A*^G.lamblia^*^CTD^ was properly assembled (Supplementary Figure S6A and B), and the DNA end flexibility was evaluated by the MNase assay. As shown in Figure 4A and B, the DNA ends of the human NCP containing H2A*^G.lamblia^*^CTD^ were substantially susceptible to MNase, as observed for the *G. lamblia* NCP. These results indicated that the *G. lamblia* H2A C-terminal region is responsible for the DNA end flexibility in the NCP. Since the interaction between the H2A C-terminal and H3 N-terminal regions is important for the DNA end binding in the canonical NCP, we tested whether the *G. lamblia* H3 is equivalently responsible for the DNA end flexibility by reconstituting an NCP containing *G. lamblia* H3–H4 and human H2A–H2B (Supplementary Figure S7A and B). As anticipated, the NCP with the *G. lamblia* H3–H4 was markedly susceptible to MNase, like the *G. lamblia* NCP, probably due to the defective interaction between *G. lamblia* H3 and human H2A (Supplementary Figure S7C). This is consistent with a weakened interaction between the H2A C-terminal and H3 N-terminal regions that induces DNA end flexibility in the *G. lamblia* NCP. This demonstrates how changes in a few key histone residues can adapt the DNA wrapping of the nucleosome with the potential to generate distinctive chromatin properties.

### The unique structural features of the H2A–H2B dimers in the *G. lamblia* NCP

**Figure 5.**
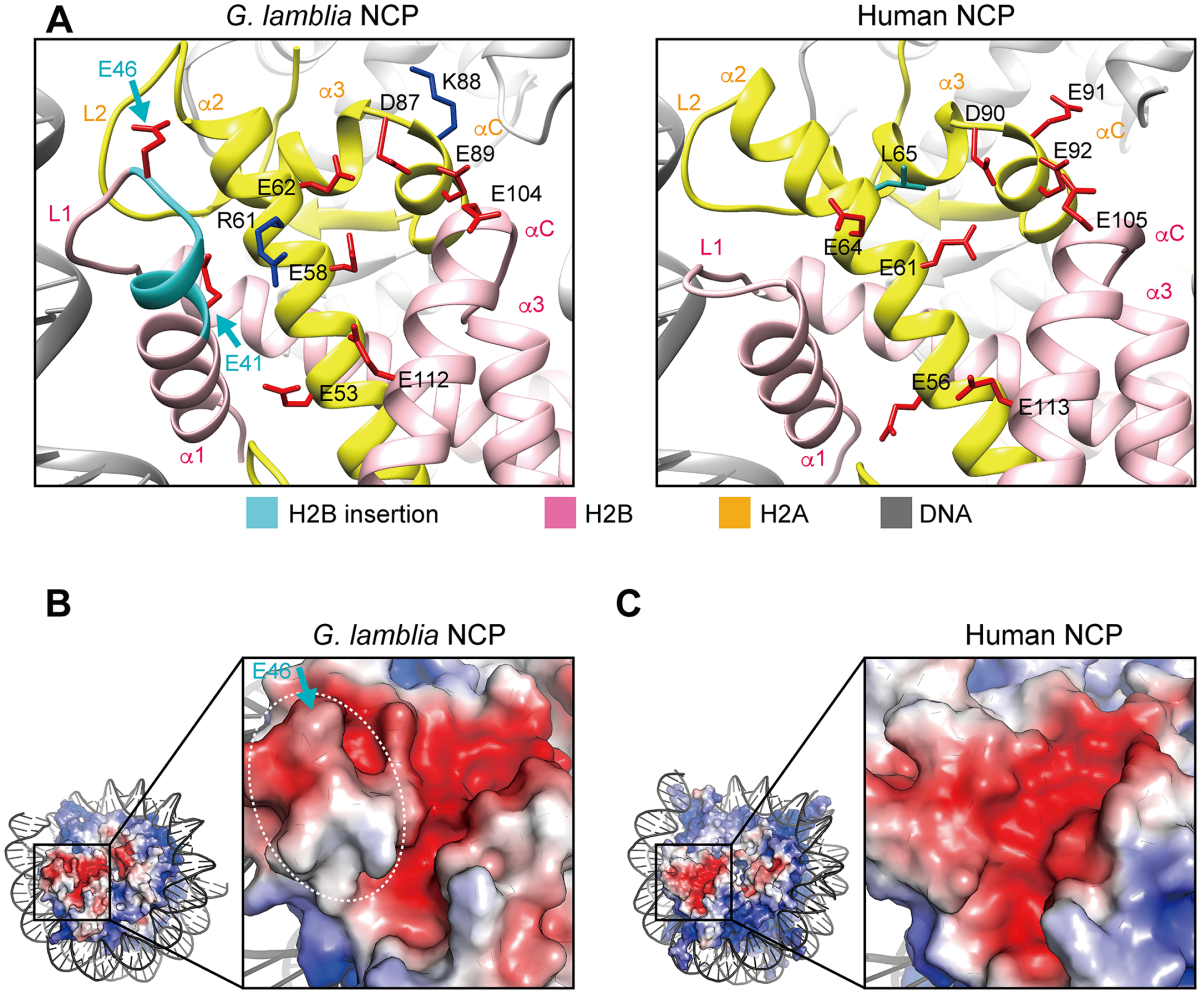
Acidic patch structure of the *G. lamblia* NCP. (**A**) Close-up views of the acidic patch region of the *G. lamblia* NCP (left panel) and the corresponding region of the human canonical NCP (PDB ID: 6R93, right panel). H2A and H2B are colored yellow and pink, respectively. The region corresponding to the unique *G. lamblia* insertion is colored cyan, and the side chains of the acidic amino acid residues in the acidic patch and the insertion are colored red. The non-conserved human H2A Leu65 residue (green) and the *G. lamblia* H2A Arg61 and Lys88 residues (dark blue) are shown. (**B**) Electrostatic potential of the acidic patch region in the *G. lamblia* NCP calculated by the APBS tool in PyMOL, with negative and positive charges in red and blue, respectively. The white dashed circle and the blue arrows indicate the ridge formed by the six residue insertion in the H2B α1 helix and the L1 loop. (**C**) Electrostatic potential of the equivalent acidic patch region in the human canonical NCP.

*G. lamblia* H2B contains a characteristic six residue insertion after position 40 (Figure 5A and Supplementary Figures S4 and S8). In the *G. lamblia* NCP structure, this insertion extends the H2B α1 helix and L1 loop. In combination with changes to 10 of the 14 C-terminal residues in the H2A α2 helix (Supplementary Figure S4), this leads to a structural difference in the *G. lamblia* H2A–H2B dimer secondary structure elements around the corresponding regions, as compared to the human H2A–H2B dimer (Figures 2A, B and 5A).

The *G. lamblia* H2A α2 and α3 regions near the H2B insertion contain a cluster of acidic amino acid residues that form the well-known acidic patch on the NCP surface (64) (Figure 5A and B). Surprisingly, the shape of this acidic patch is substantially different from that of the human NCP (Figure 5B and C), because the *G. lamblia* H2B insertion forms a ridge that deepens the acidic patch (Figure 5B). Furthermore, the human H2A residues Glu64, Leu65, and Glu91 are replaced by the *G. lamblia* H2A Arg61, Glu62, and Lys88, respectively (Figure 5A and Supplementary Figure S4). These structural differences and substitutions in the *G. lamblia* H2A and H2B dramatically affect the morphology of the acidic patch in the NCP (Figure 5B and C).

**Figure 6.**
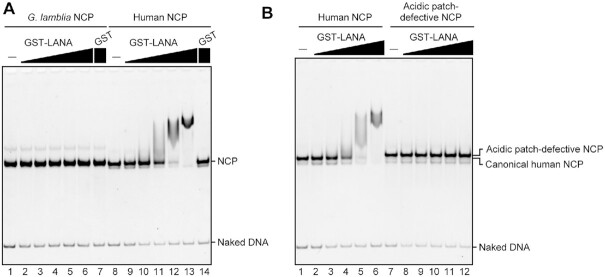
LANA acidic patch binding peptide does not bind to the *G. lamblia* NCP. (**A**) GST-LANA (1, 2, 4, 8, 16 and 32 μM) or GST (32 μM) were incubated with the human NCP (0.5 μM) or the *G. lamblia* NCP (0.5 μM). The complex formation was analyzed by non-denaturing PAGE with ethidium bromide staining. (**B**) GST-LANA (1, 2, 4, 8, 16 and 32 μM) was incubated with the human NCP (0.5 μM) or the acidic patch-defective NCP (0.5 μM) containing a 145 bp Widom 601 DNA, as in A. An independent replicate experiment is shown in Supplementary Figure S9.

The H2A–H2B acidic patch functions as a recognition site for nucleosome binding proteins (64,65). We prepared the well-known LANA acidic patch binding peptide (66,67) as a GST-fused protein (Supplementary Figure S9A), and performed the electrophoretic mobility shift assay. The *G. lamblia* NCP was quite defective in binding to the LANA peptide (Figure 6A, lanes 1–6), whereas it bound efficiently to the human NCP (Figure 6A, lanes 8–13), but not to an acidic patch-defective NCP mutant (68) (Figure 6B, lanes 7–12). These results demonstrated that the unique acidic patch conformation of the *G. lamblia* NCP can lead to distinctive protein binding properties.

The acidic patch could also regulate the activities of chromatin remodelers (69,70). *G. lamblia* contains only a minimal repertoire of Snf2 family chromatin remodeling proteins and chromatin interacting proteins, which may have provided an opportunity for the specialization of the chromatin properties (71,72). The unusual acidic patch conformation in the *G. lamblia* NCP may also be an interesting target for the future development of inhibitors for this parasite.

### Higher order structure of the *G. lamblia* nucleosome array

**Figure 7.**
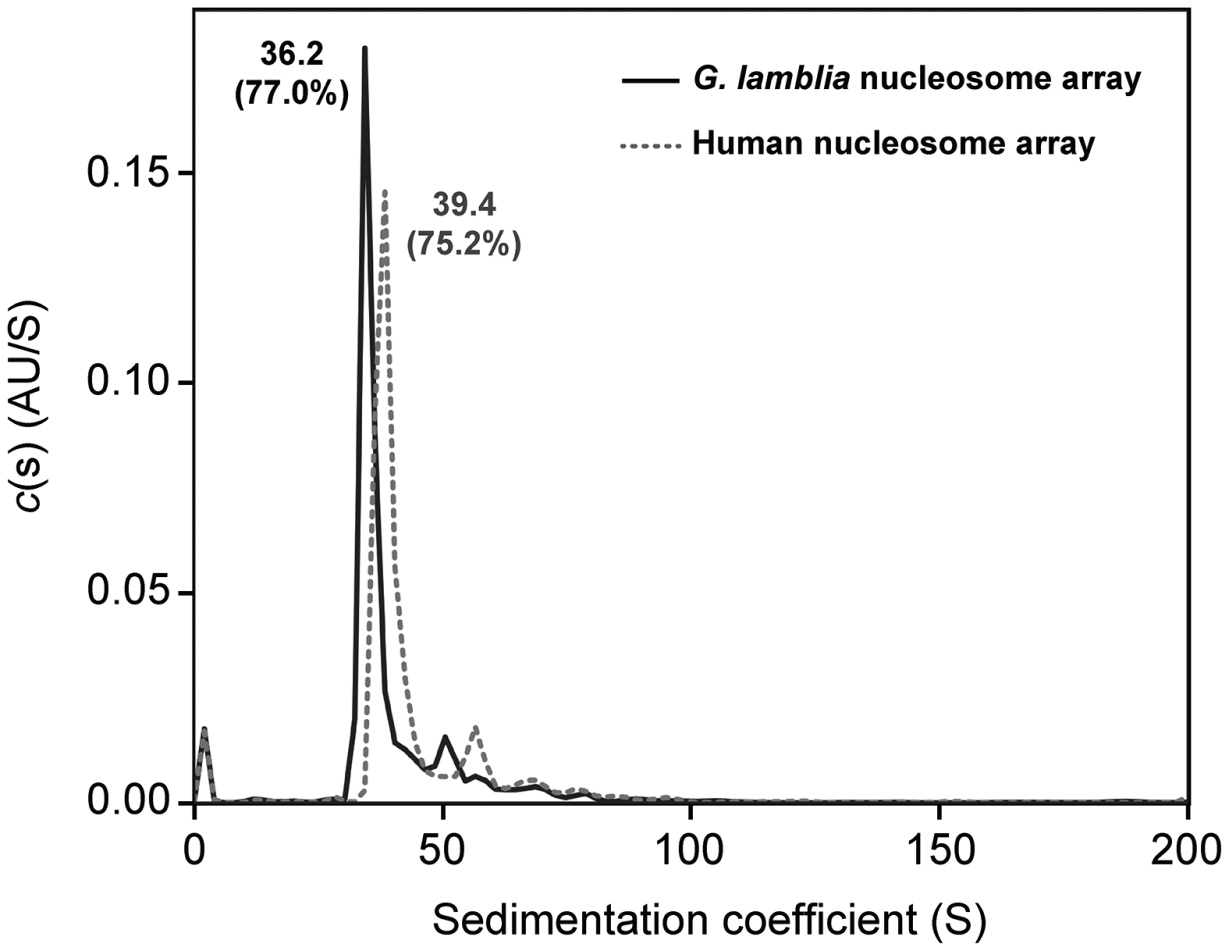
Analytical ultracentrifugation sedimentation velocity analysis. The nucleosome arrays containing the human and *G. lamblia* histones were subjected to analytical ultracentrifugation at 22 000 rpm, with scanning at 260 nm, in 10 mM Tris–HCl buffer (pH 7.5). The collected data were analyzed using the continuous c(s) distribution model with the SEDFIT software. An independent replicate experiment is shown in Supplementary Figure S10D.

The acidic patch binds the H4 N-terminal tail of the nearby nucleosome, suggesting a role in chromatin compaction through the nucleosome-nucleosome interactions (5,64,65,73). The flexibility of the DNA entry/exit regions can also affect the chromatin compaction. Accordingly, we performed the sedimentation velocity assay by analytical ultracentrifugation with nucleosome arrays (46) containing twelve *G. lamblia* or human canonical nucleosomes (Supplementary Figure S10). As shown in Figure 7, the *S* value of the *G. lamblia* nucleosome array was 36.2, while the value for the human nucleosome array under the same experimental conditions was 39.4. This indicates that the *G. lamblia* nucleosome array may be more open than the human nucleosome array. This may be due to the entry/exit DNA end flexibility observed in the *G. lamblia* NCP and/or for the unique inter-nucleosomal interactions between the acidic patch and the H4 N-terminal tail in the *G. lamblia* nucleosome array.

### Stability of the *G. lamblia* NCP

**Figure 8.**
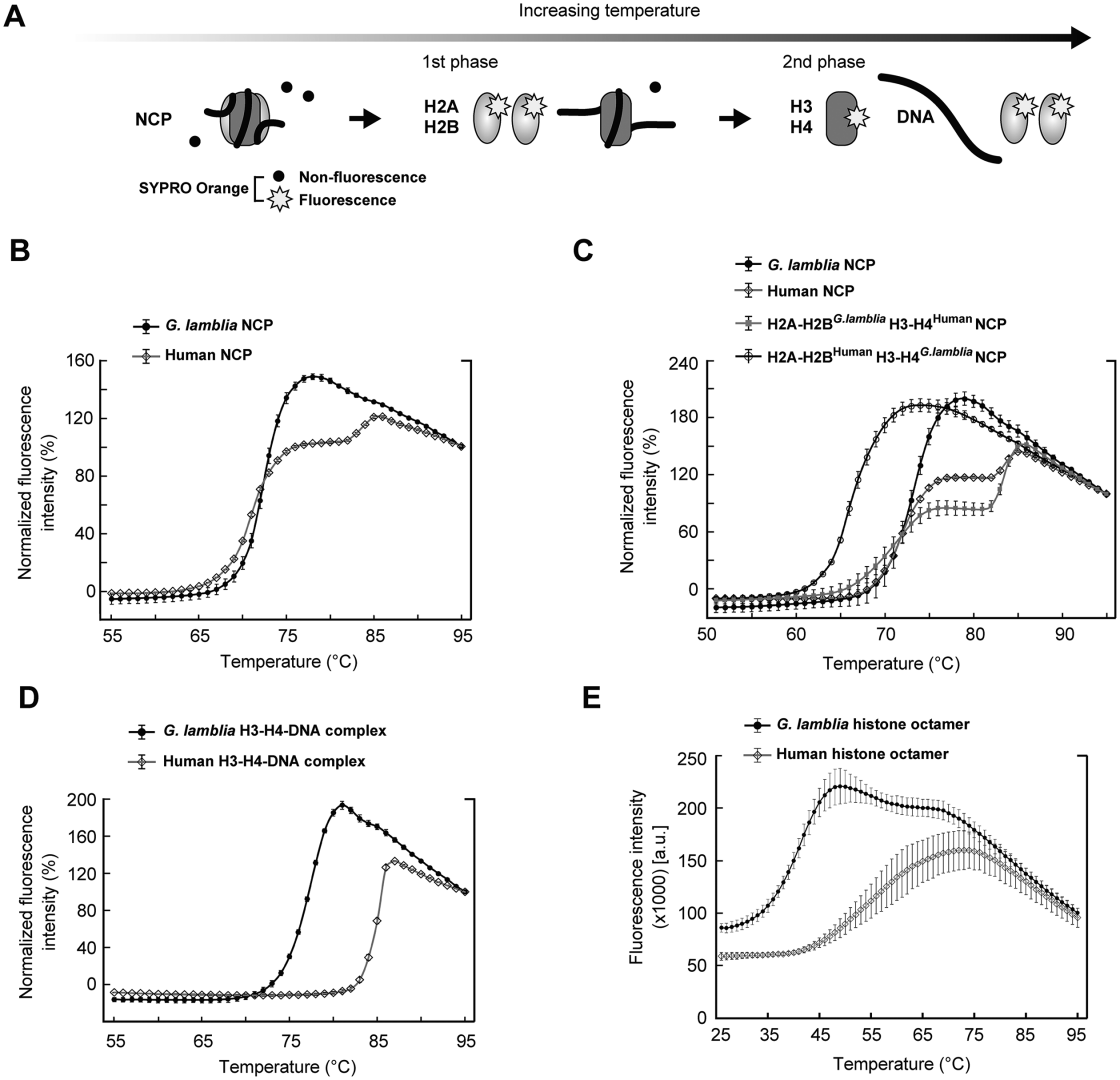
Thermal stabilities of the *G. lamblia* NCP, H3–H4–DNA complex, and histone octamer. (**A**) Schematic representation of the thermal stability assay. (**B**) Thermal denaturation plots of *G. lamblia* and human canonical NCPs, with error bars representing standard deviations (*n* = 3). (**C**) Thermal denaturation plots of the NCPs containing the *G. lamblia* H2A–H2B and human H3–H4 histones (H2A–H2B*^G.lamblia^*H3–H4^Human^), and the human H2A–H2B and *G. lamblia* H3–H4 histones (H2A–H2B^Human^H3–H4*^G.lamblia^*), with error bars representing standard deviations (*n* = 3). (**D**) Thermal denaturation plots of the *G. lamblia* and human H3–H4–DNA complexes, with error bars representing standard deviations (*n* = 3). (**E**) Thermal denaturation plots of the *G. lamblia* and human histone octamers, with error bars representing standard deviations (*n* = 3).

Finally, we tested the stability of the *G. lamblia* NCP by a thermal stability assay, in which the thermal denaturation of the NCP can be estimated according to the fluorescence signal from SYPRO Orange bound to denatured histones (Figure 8A) (48). The human canonical NCPs denatured with a biphasic profile, in which the first and second phases correspond to the H2A–H2B and H3–H4 dissociations, respectively (Figure 8A and B), as defined previously (48). In contrast, the denaturation of the *G. lamblia* NCP was mainly represented by an effectively monophasic profile aligned to the first phase (Figure 8B). Interestingly, the NCP with *G. lamblia* H2A–H2B and human H3–H4 exhibited a similar thermal denaturation profile to the human NCP (Figure 8C). In contrast, the NCP with human H2A–H2B and *G. lamblia* H3–H4 was extremely unstable as compared to the human NCP (Figure 8C), suggesting that the *G. lamblia* H3–H4 surfaces are responsible for the instability of the *G. lamblia* NCP. The denaturation profile of the *G. lamblia* NCP is quite similar to that of the human NCP containing the *Leishmania major* histone H3 (LmaH3), where LmaH3 weakens the histone-DNA association in the NCP (74). To test *G. lamblia* H3–H4 binding to DNA, we performed the thermal stability assay with the H3–H4–DNA complex. As shown in Figure 8D, the *G. lamblia* H3–H4–DNA complex dissociated at a substantially lower temperature than the human canonical H3–H4–DNA complex, suggesting that *G. lamblia* histones form an unstable NCP due to the weakened DNA association by the H3–H4 complex.

To evaluate the histone–histone interactions in the *G. lamblia* histone octamer, we reconstituted the histone octamers with the *G. lamblia* and human histones in the absence of DNA (Supplementary Figure S11). H3 and H4 were mixed with excess amounts of H2A and H2B under denaturing conditions, and the histone complexes were assembled by dialysis against buffer containing 2 M NaCl. Both the *G. lamblia* and human histone octamers efficiently formed under these conditions, and could be purified by gel filtration chromatography (Supplementary Figure S11). The thermal stability assay with the histone octamers showed that the fluorescence intensity of the *G. lamblia* histone octamer rapidly increased in the temperature range of 28–49°C, which is considerably lower than that of the human histone octamer, in the range of 39–74°C (Figure 8E). These results are consistent with the unstable nature of the *G. lamblia* NCP and indicate that the combined sequence divergence of the *G. lamblia* histone octamer makes it less stable and potentially more dynamically accessible than the orthologous human histone octamer.

## CONCLUSION

We have determined the cryo-EM structure of the *G. lamblia* NCP, which revealed that it retains the similar overall structural organization as metazoan NCPs despite the very high degree of histone sequence divergence. However, we also observed that histone sequence variation gives rise to unique structural and biochemical features in the *G. lamblia* NCP. The DNA and histone regions near the DNA entry-exit sites of the *G. lamblia* NCP are remarkably flexible, and the morphology of the *G. lamblia* NCP acidic patch is profoundly different. In addition, the *G. lamblia* NCP is quite unstable as compared to the human canonical NCP, probably due to the weakened binding of the H3–H4 complex to both H2A–H2B and DNA. These structural and physical characteristics of the *G. lamblia* NCP illustrate the potential for the diversity of chromatin architecture and function, because *G. lamblia* may be less constrained by the complex interdependencies of chromatin interacting factors. The NCP structure may be useful in future drug design and discovery to treat infections by this important parasite.

## DATA AVAILABILITY

The cryo-EM structure and the atomic model of the *G. lamblia* NCP have been deposited in the Electron Microscopy Data Bank under the accession code EMD-30591, and in the Protein Data Bank under the accession code PDB ID 7D69.

## Supplementary Material

gkab644_Supplemental_File
