# Supplementary material for: Cryo-EM structure of the nucleosome core particle containing Giardia lamblia histones
Source: Nucleic Acids Res. 2021 Aug 5;49(15):8934–46. doi: 10.1093/nar/gkab644 (PMC8421212; doi:10.1093/nar/gkab644)
Supplement: gkab644_Supplemental_File [file gkab644_supplemental_file.pdf]

Supplementary Figure S1

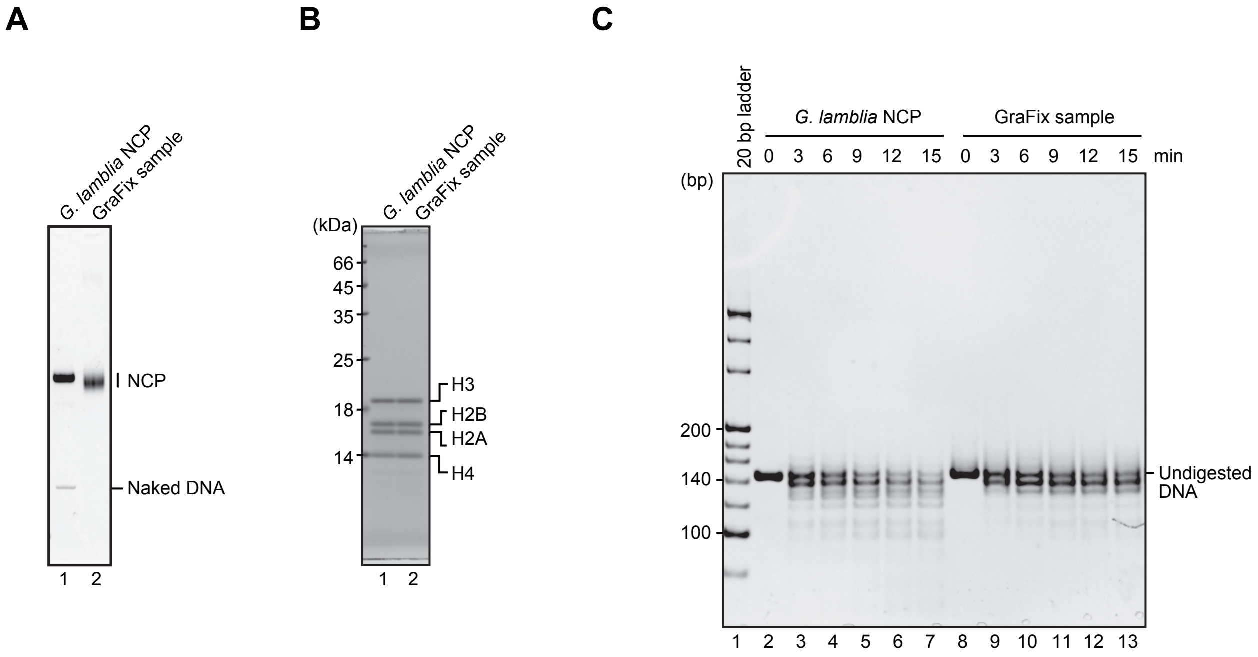

Supplementary Figure S1

Preparation of the NCP containing the *G. lamblia* histones. (A) The NCP containing the *G. lamblia* histones was analyzed by non-denaturing polyacrylamide gel electrophoresis, followed by ethidium bromide staining. Lanes 1 and 2 indicate the *G. lamblia* NCPs without and with fixation by the GraFix method, respectively. (B) The *G. lamblia* histones in the NCP were analyzed by SDS-polyacrylamide gel electrophoresis, followed by Coomassie Brilliant Blue staining. Lanes 1 and 2 indicate the *G. lamblia* NCPs without and with fixation by the GraFix method, respectively. The sample with fixation (lane 2) was de-crosslinked before the SDS-polyacrylamide gel electrophoresis analysis. (C) The NCPs (1.4 μg for DNA) containing the *G. lamblia* histones were incubated with 0.7 units of MNase at 37°C, in a reaction solution containing 40 mM Tris-HCl (pH 8.0), 25 mM NaCl, 2.5 mM $CaCl_2$, and 1.9 mM dithiothreitol. The reactions were stopped by adding an aliquot (10 μl) to 5 μl of a deproteinization solution, consisting of 20 mM Tris-HCl (pH 8.0), 20 mM EDTA, 0.1% SDS, and 0.5 mg/ml proteinase K. The reaction products were analyzed by non-denaturing polyacrylamide gel electrophoresis, followed by ethidium bromide staining. Lanes 2-7 and lanes 8-13 indicate the *G. lamblia* NCPs without and with fixation by the GraFix method, respectively.

Supplementary Figure S2

A

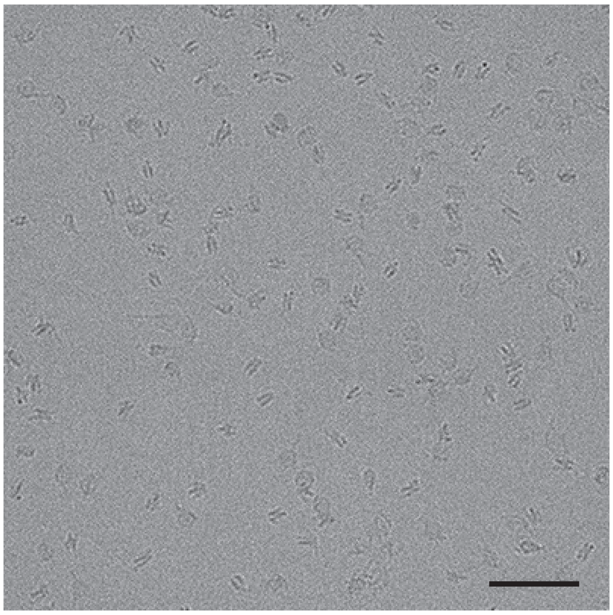

B

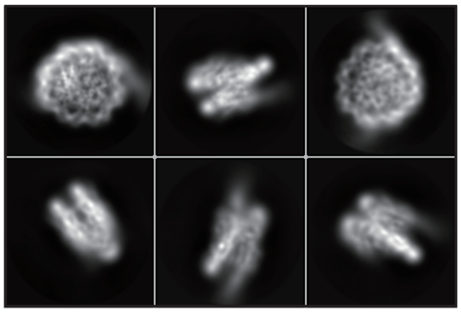

C

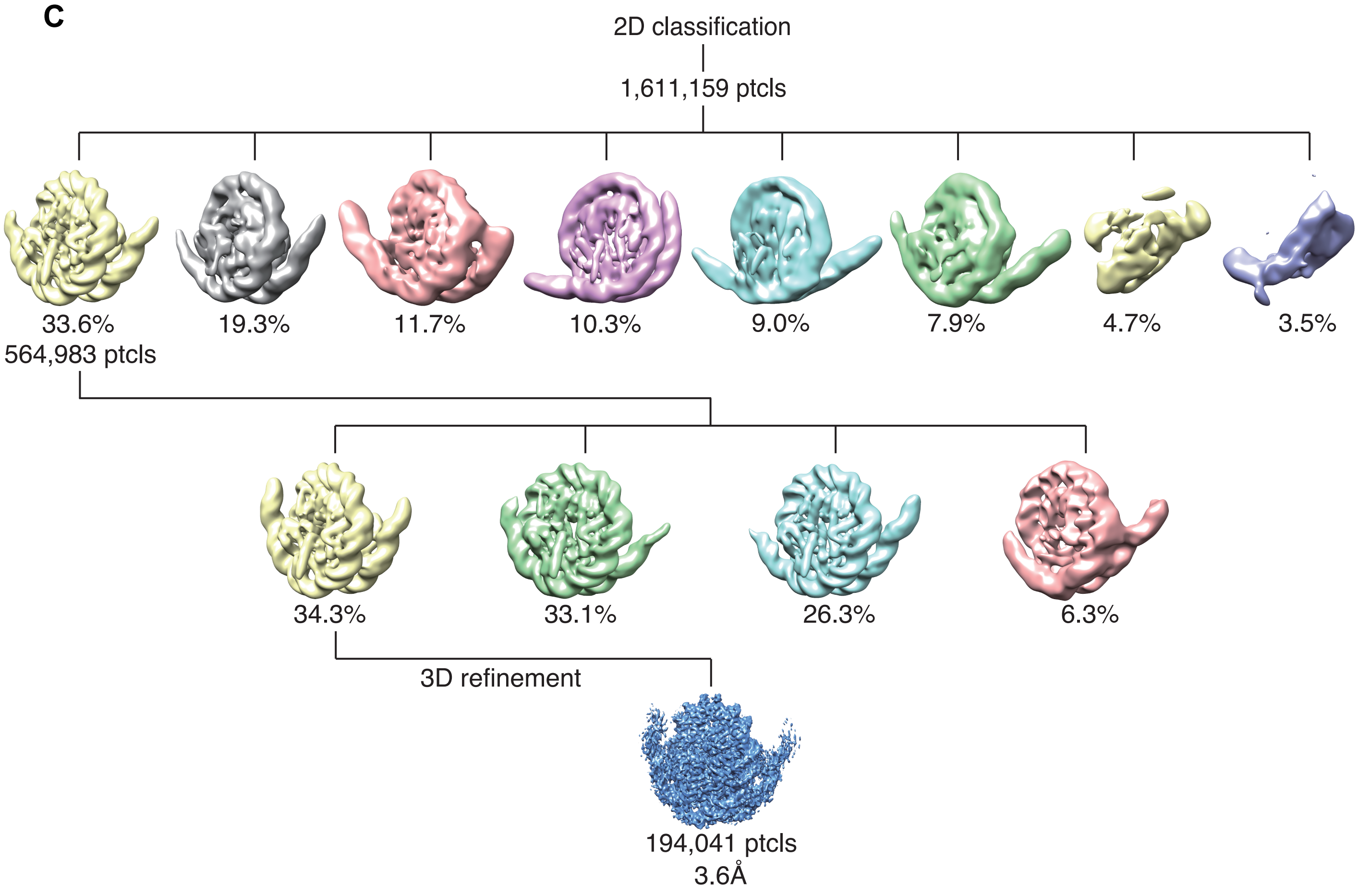

Supplementary Figure S2

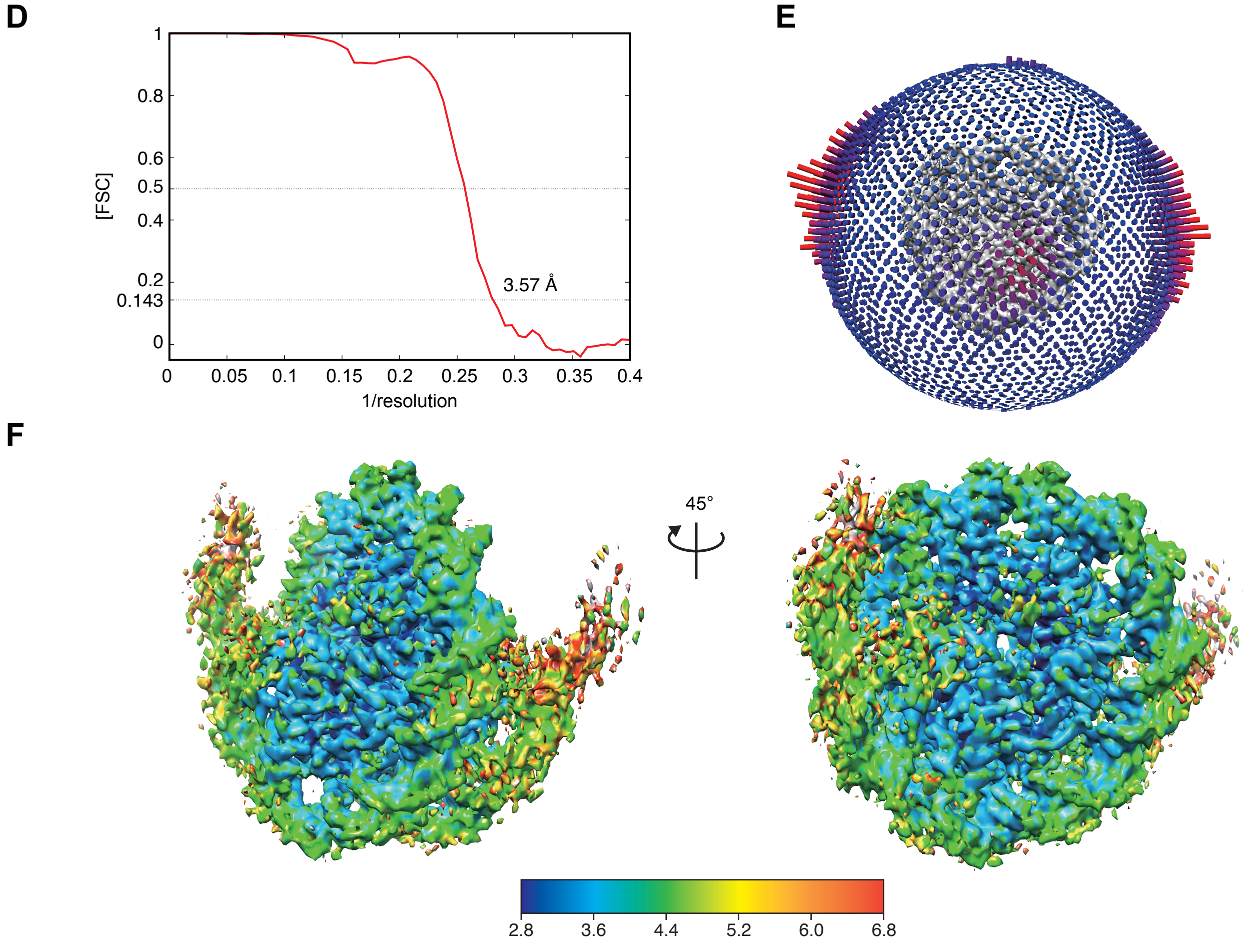

Supplementary Figure S2

Cryo-EM analysis of the *G. lamblia* NCP. (A) Representative micrograph of the *G. lamblia* NCP. Scale bar, 50 nm. (B) Representative 2D class averages of the *G. lamblia* NCP. Box size, 16.8 nm. (C) Flow chart of the cryo-EM data processing procedure. The percentage of the particle numbers for each classification step, as well as the particle number and resolution of the refinement 3D structure, are shown at the bottom. (D) Fourier Shell Correlation (FSC) calculated between independently refined image datasets of the *G. lamblia* NCP. The overall resolution of the *G. lamblia* NCP is 3.57 Å (FSC=0.143). (E) Euler angle distribution map of all particles used in the 3D reconstitution of the *G. lamblia* NCP. (F) Local resolution map of the *G. lamblia* NCP, showing the range between 2.8 to 6.8 Å.

Supplementary Figure S3

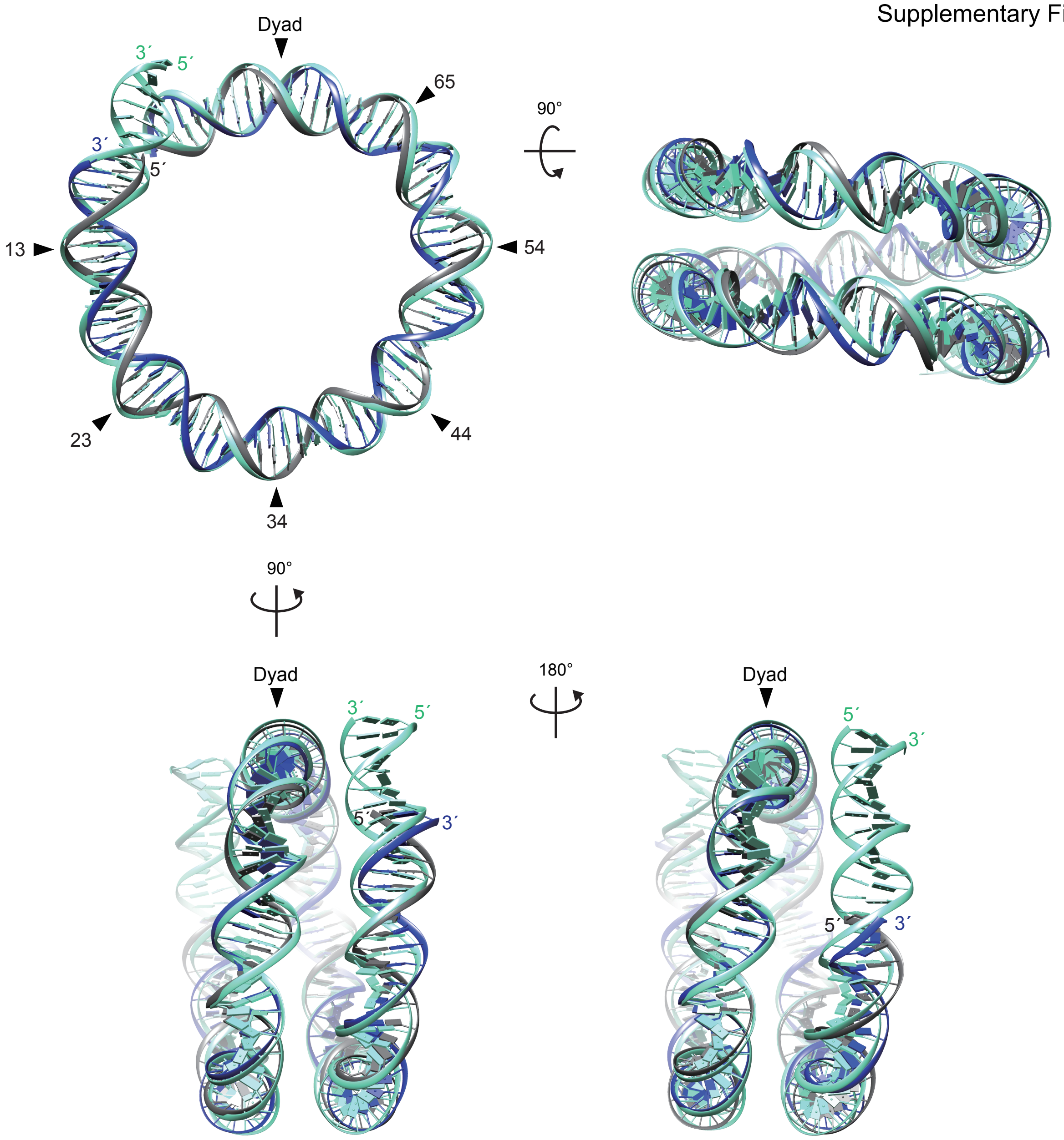

Supplementary Figure S3

DNA structure of the *G. lamblia* NCP. The DNA structure of the *G. lamblia* NCP was superimposed on the canonical NCP (PDB ID: 3UT9) with the identical 601L DNA sequence. The left panel displays the half side of the nucleosomal DNA. The DNA strands in the *G. lamblia* NCP are colored gray and dark blue. The DNA strands in the canonical NCP are colored light blue.

Supplementary Figure S4

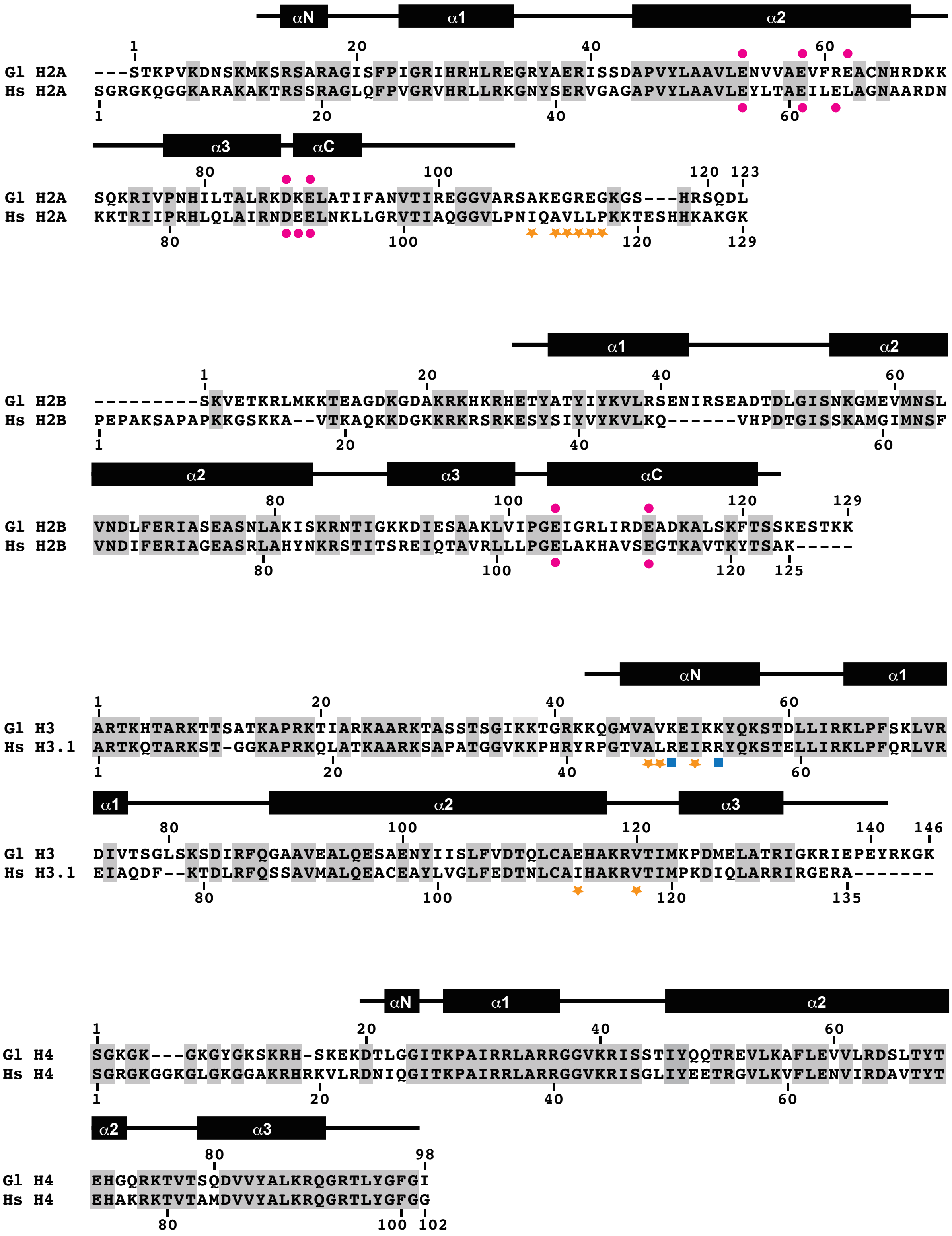

Supplementary Figure S4

Supplementary Figure S4

Sequence alignments of the *G. lamblia* and human canonical histones. Conserved residues are shown with a gray background, and the *G. lamblia* helical assignments are above the sequence. The amino acid residues related to the hydrophobic core formation between H3 and the H2A C-terminal region are indicated by orange stars. The acidic amino acid residues involved in the H2A-H2B acidic patch are indicated by magenta dots. The arginine residues involved in the DNA flexibility around the entry-exit regions of the human NCP are indicated by blue squares.

Supplementary Figure S5

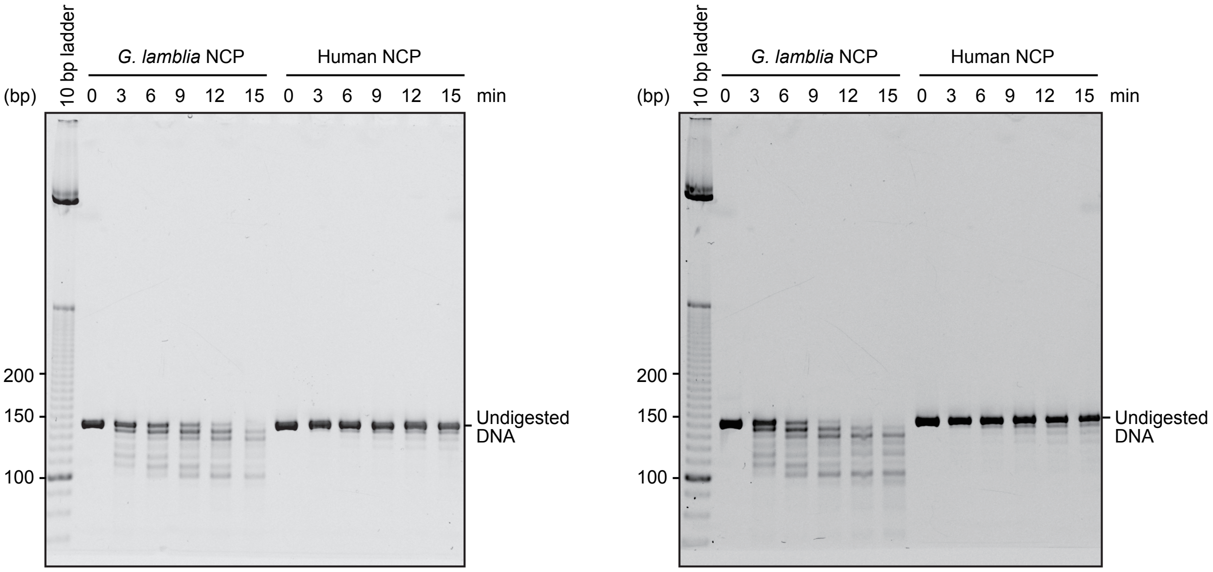

Supplementary Figure S5

Replicated experiments for the MNase susceptibility assay shown in Fig. 3D.

Supplementary Figure S6

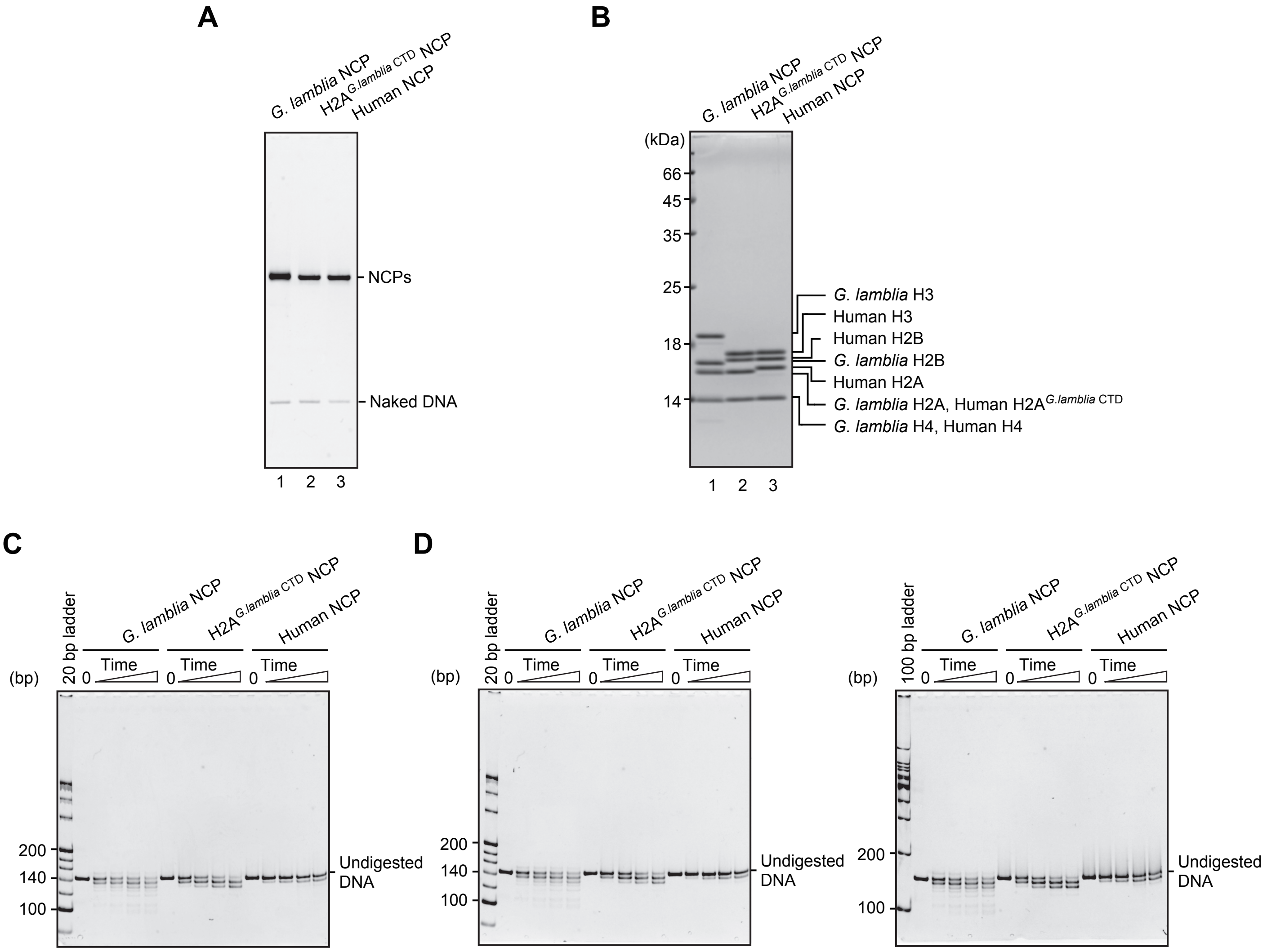

Supplementary Figure S6

Sample preparation and MNase assay with H2A$^{G. lamblia\ \mathrm{CTD}}$. (A) The reconstituted NCPs were analyzed by non-denaturing polyacrylamide gel electrophoresis, followed by ethidium bromide staining. Lanes 1, 2, and 3 indicate the *G. lamblia* NCP, the H2A$^{G. lamblia\ \mathrm{CTD}}$ NCP, and the human NCP, respectively. (B) The NCPs were analyzed by SDS-polyacrylamide gel electrophoresis, followed by Coomassie Brilliant Blue staining. Lanes 1, 2, and 3 indicate the *G. lamblia* NCP, the H2A$^{G. lamblia\ \mathrm{CTD}}$ NCP, and the human NCP, respectively. (C) A full image of the MNase assay shown in Fig. 4A. (D) Replicated experiments for the MNase assay shown in Fig. 4A.

Supplementary Figure S7

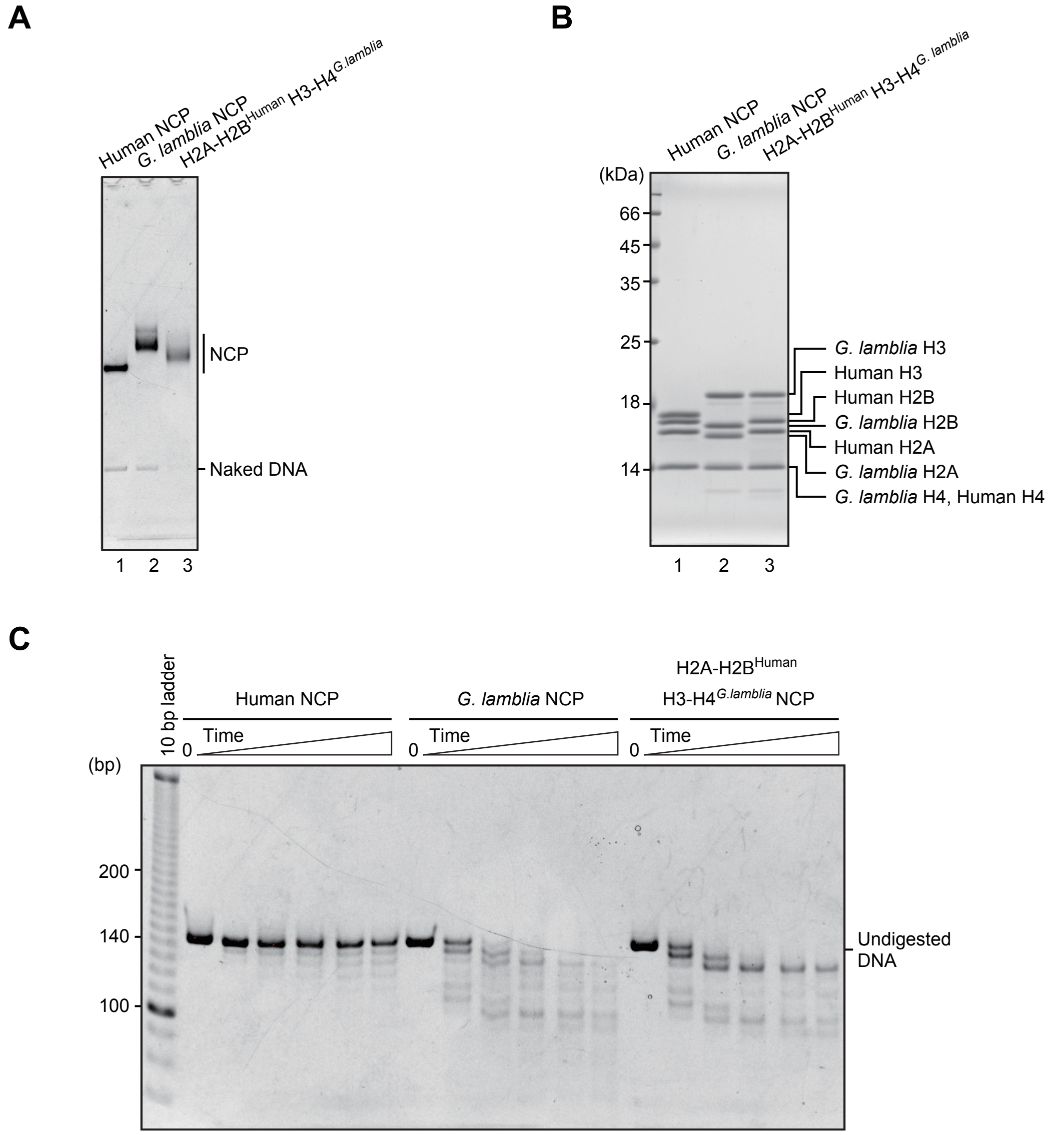

Supplementary Figure S7

Sample preparation and MNase assay of the chimeric NCPs with the *G. lamblia* and human histones. (A) The NCPs were analyzed by non-denaturing polyacrylamide gel electrophoresis, followed by ethidium bromide staining. Lanes 1, 2, and 3 indicate the human NCP, the *G. lamblia* NCP, and the NCP with human H2A-H2B and *G. lamblia* H3-H4, respectively. (B) The NCPs were analyzed by SDS-polyacrylamide gel electrophoresis, followed by Coomassie Brilliant Blue staining. Lanes 1, 2, and 3 indicate the human NCP, the *G. lamblia* NCP, and the NCP with human H2A-H2B and *G. lamblia* H3-H4, respectively. (C) A full image of the MNase assay with the human NCP, the *G. lamblia* NCP, and the NCP with human H2A-H2B and *G. lamblia* H3-H4. The experiments were repeated, and the reproducibility was confirmed.

Supplementary Figure S8

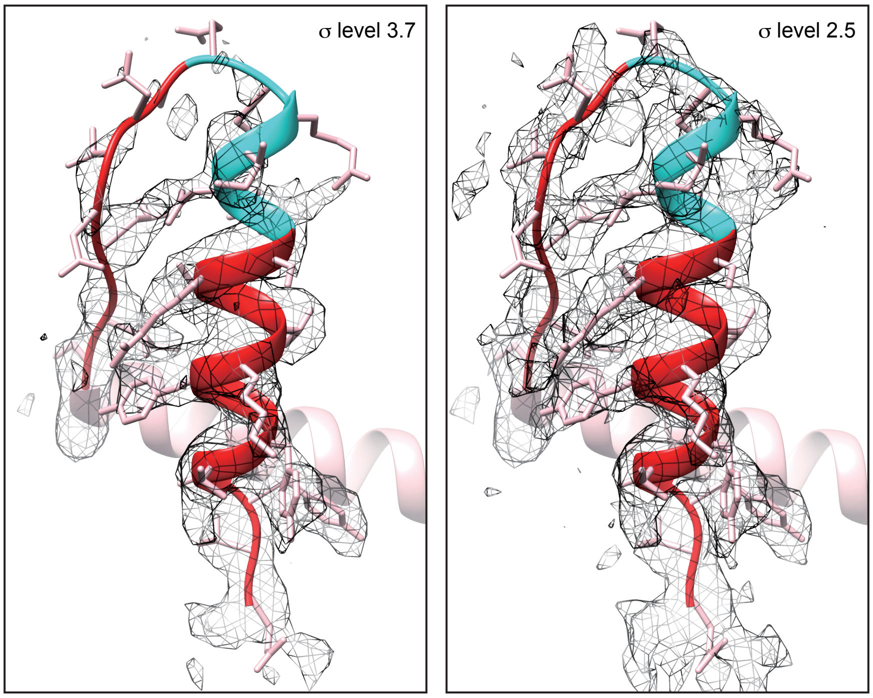

Supplementary Figure S8

The insertion of the six amino acid residues in the *G. lamblia* H2B. The cryo-EM maps for the H2B α1 helix and L1 loop regions containing the *G. lamblia*-specific insertion are presented at the indicated sigma levels.

Supplementary Figure S9

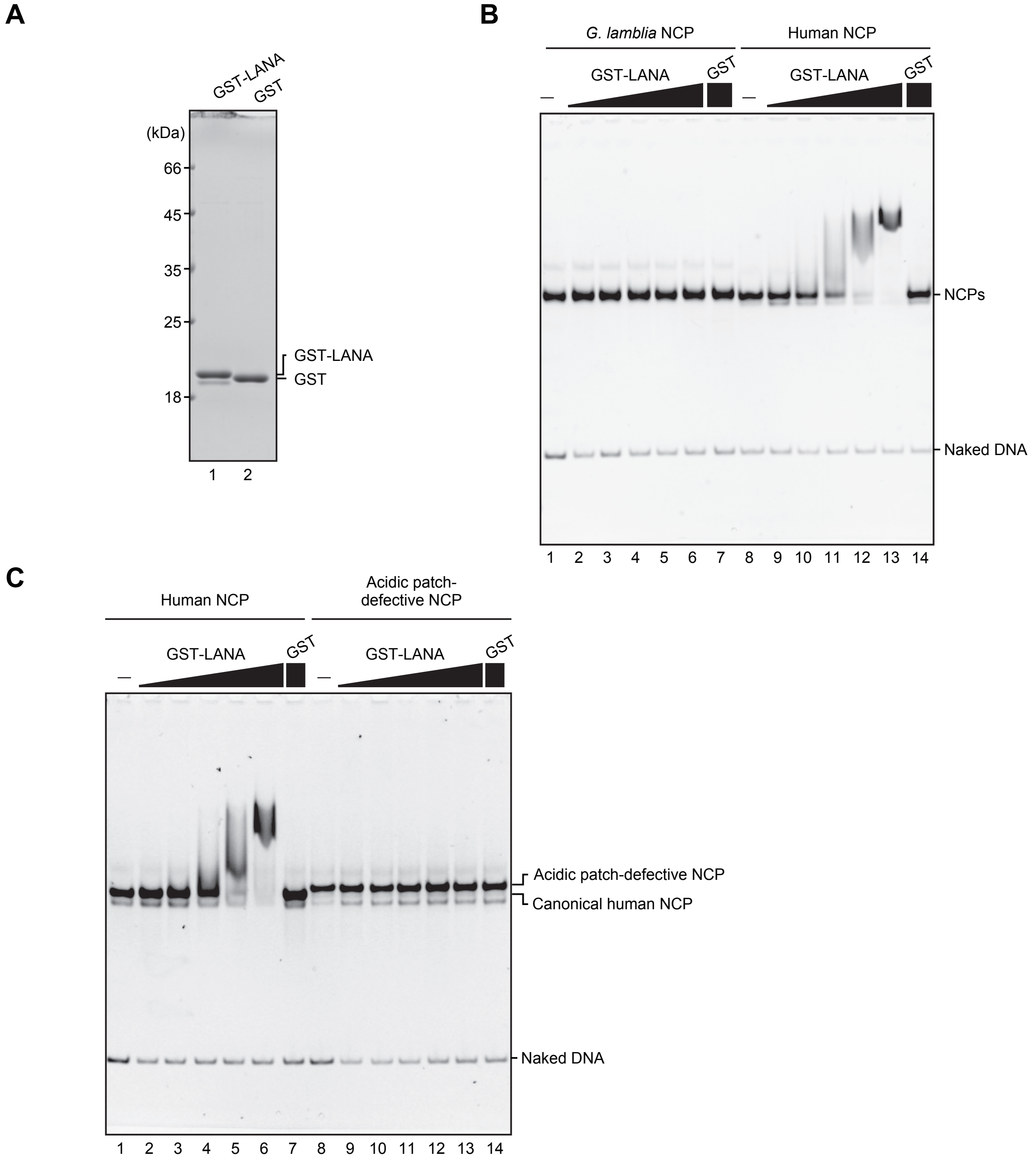

Supplementary Figure S9

The interaction between the NCPs and the LANA peptide. (A) Purified proteins were analyzed by SDS-polyacrylamide gel electrophoresis with Coomassie Brilliant Blue staining. Lanes 1 and 2 indicate glutathione S-transferase (GST)-fused LANA peptide containing residues 1-23, and GST, respectively. (B) Replicated experiment for the electrophoretic gel mobility shift assay shown in Fig. 6(A). (C) Replicated experiment for the electrophoretic gel mobility shift assay shown in Fig. 6(B).

Supplementary Figure S10

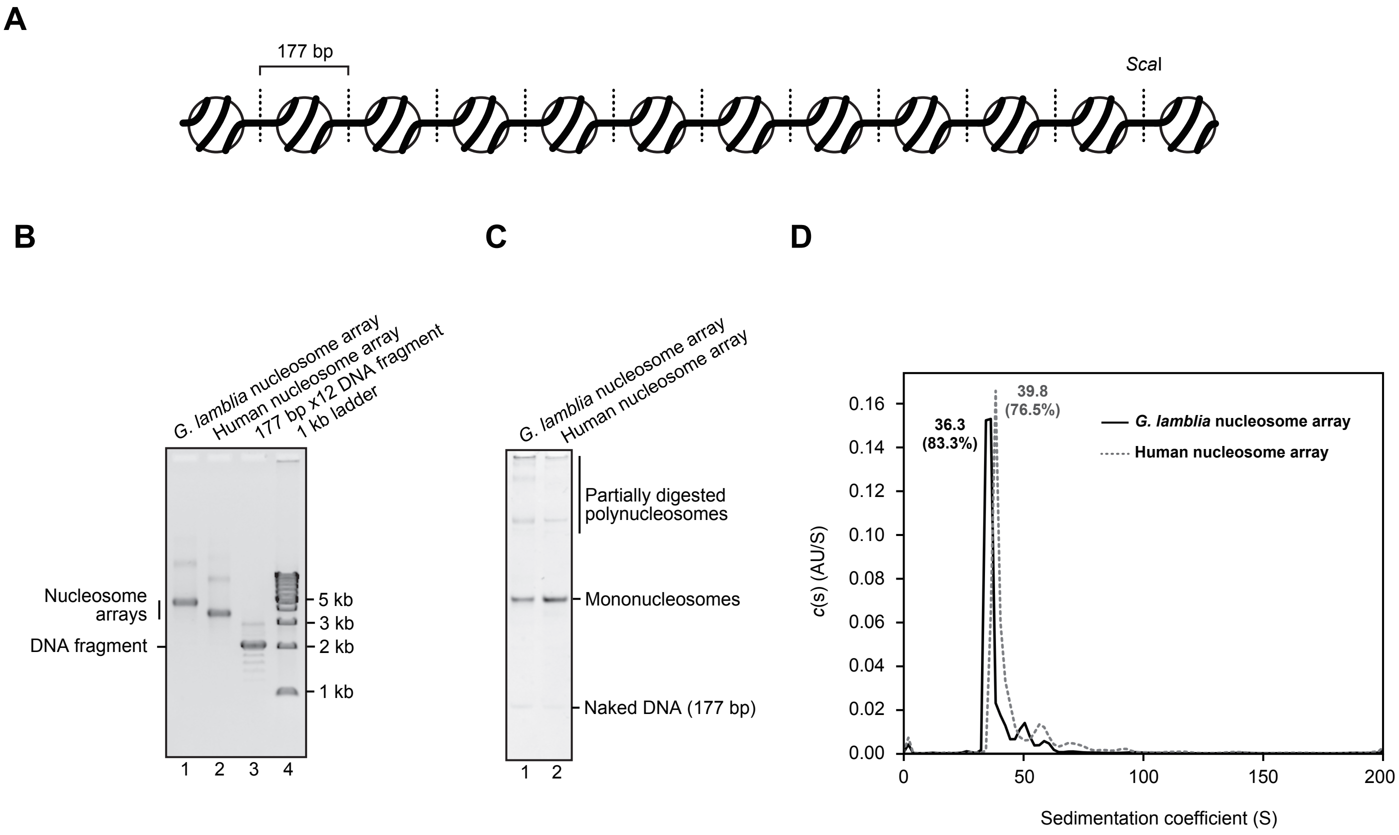

Supplementary Figure S10

Analytical ultracentrifugation sedimentation velocity experiments. (A) Schematic representation of the nucleosome array. (B) The nucleosome arrays and DNA fragment were analyzed by electrophoresis on a non-denaturing 0.7% agarose gel in 0.2xTBE buffer. Lanes 1 and 2 indicate the nucleosome arrays containing the *G. lamblia* and human histones, respectively. Lane 3 indicates the DNA fragment containing twelve tandem repeats of a 177 base-pair 601 sequence. (C) Nucleosome occupancy in the *G. lamblia* and human nucleosome arrays. The nucleosome arrays were digested by a restriction enzyme, *Sca*I, and the resulting mononucleosome products were analyzed by non-denaturing 5% polyacrylamide gel electrophoresis in 1xTBE buffer. Lanes 1 and 2 indicate the digested nucleosome arrays containing the *G. lamblia* and human histones, respectively. The nucleosome occupancy can be estimated by the amount of the resulting naked DNA. (D) Replicated experiment for the analytical ultracentrifugation sedimentation velocity experiment shown in Fig. 7. The *S* values of the *G. lamblia* and human nucleosome arrays were 36.3 and 39.8, respectively, under the same experimental conditions.

Supplementary Figure S11

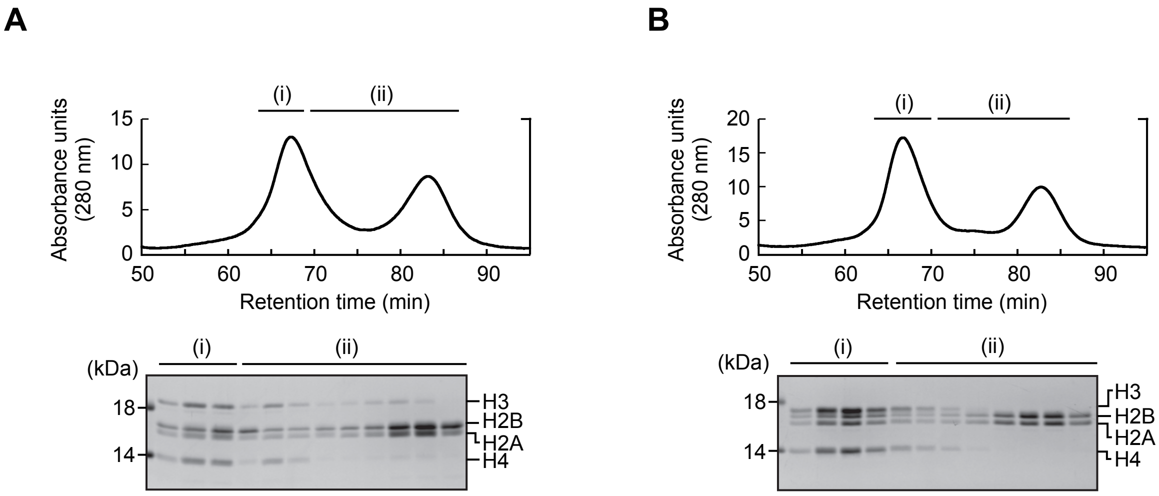

Supplementary Figure S11

Histone octamer formation by *G. lamblia* histones. The *G. lamblia* histones H2A, H2B, H3, and H4 were mixed at a 1.0:1.0:1.2:1.2 molar ratio under denaturing conditions in 50 mM Tris-HCl buffer (pH 7.5), containing 7 M guanidine hydrochloride and 20 mM 2-mercaptoethanol. Complexes were assembled by dialysis against 20 mM Tris-HCl buffer (pH 7.5), containing 2 M NaCl, 1 mM EDTA, and 5 mM 2-mercaptoethanol. The complexes were then fractionated by size exclusion chromatography on a Hiload 16/60 Superdex 200 prep grade column. (A) Elution profiles of the *G. lamblia* histone complex (upper panel). Histone compositions of the peak fractions, (i) and (ii), analyzed by 18% SDS-polyacrylamide gel electrophoresis with Coomassie Brilliant Blue staining (bottom panel). (B) Elution profiles of the human canonical histone complex (upper panel). Histone compositions of the peak fractions, (i) and (ii), analyzed by 18% SDS-polyacrylamide gel electrophoresis with Coomassie Brilliant Blue staining (bottom panel).
